# Discovery of two new species of *Crotalaria* (Leguminosae, Crotalarieae) from Western Ghats, India

**DOI:** 10.1371/journal.pone.0192226

**Published:** 2018-02-15

**Authors:** Shabir A. Rather, Shweta Subramaniam, Shagun Danda, Arun K. Pandey

**Affiliations:** Plant Systematics Laboratory, Department of Botany, University of Delhi, Delhi, India; Indiana University Bloomington, UNITED STATES

## Abstract

Two new species of Fabaceae-Papilionoideae are described and illustrated. *Crotalaria suffruticosa* from Karul Ghat region of Maharashtra is morphologically close to *C*. *albida* and *C*. *epunctata*. *C*. *multibracteata* from Panhala region of Maharashtra resembles *C*. *vestita*. *C*. *suffruticosa* differs from *C*. *albida* and *C*. *epunctata* in its habit, leaf, inflorescence, callosity, keel type, stigma, style morphology and number of seeds/pod. To test if the new species differ from their morphologically most similar species, we measured various traits and performed a Principal Component Analysis (PCA). This analysis shows that the new species differs from similar species in gross morphology for several diagnostic traits and showed correlations between the variables or distance among groups and estimated the contribution of each character. Phylogenetic analyses were also conducted based on nuclear (ITS) and plastid (*matK*) markers. The analyses revealed nucleotide differences between the new species and their close allies attributing to their distinctiveness. A map and key including all species of *Crotalaria* from Maharashtra state are provided. Conservation status of the two new species have also been assessed.

## Introduction

The Crotalarieae (Benth.) Hutch., (Fabaceae) is the largest tribe in the genistoid alliance (containing 51% of genistoid legumes) and comprises 16 genera and 1204 species [1–5]. More than half of the diversity of the tribe belongs to the genus *Crotalaria* L., with 702 species [6–7]. Recent molecular work has provided profound insights into generic and specific relationships and better understanding of the group in the tribe Crotalarieae and the genus *Crotalaria*, thereby establishing the monophyly of the genus [3, 5–6, 8]. An infrageneric classification of *Crotalaria* was attempted by Le Roux et al. [6], based on molecular phylogenetic data, which brought significant advances in the understanding of the infrageneric classification, redefined and complemented the previous classification given by Polhill in 1982 [9]. In India, the revisionary work on the genus *Crotalaria* was undertaken by Ansari [10] to which further data was incorporated by Subramaniam et al. [8]. The genus *Crotalaria* is distributed in tropical and sub-tropical regions of the world. The species of *Crotalaria* exhibits great diversity of habit and ecological preferences. The genus chiefly colonizes open grasslands and forest edges. There are both annual and perennial species, the habit including prostrate or erect herbs, under-shrubs, robust shrubs and rarely trees (e.g., *C*. *agatiflora* Schweinf. ex Engl.) [8–9].

The Genus *Crotalaria* is pantropical and the maximum species diversity occurs in Africa and Madagascar (ca. 540 species) with further expansions into South America (64 species), and North America (34 species) [6, 11–12]. In Asia, India hosts the maximum number of species with radiations in Pakistan (15 species) [13] and South East Asia which includes: Indonesia, Laos, Philippines, Vietnam, Cambodia, Malaysia, Thailand, Myanmar, Brunei, Singapore, Timor-leste, Andaman and Nicobar Islands (with 105 species) [14]. In India, the genus is represented by 85 species [15–16], of which 73 species are restricted to Peninsular India (Karnataka, Andhra Pradesh, Kerala, and Tamil Nadu). The species are concentrated mainly in the belt of Western Ghats (the Sahyadri) starting from Maharashtra, south of the Tapti river, and running approximately 1,600 km through the states of Maharashtra, Goa, Karnataka, Kerala and Tamil Nadu ending at Kanyakumari, at the southern tip of India [10–12, 15]. Of the total species of *Crotalaria* naturally occurring in India, 47% are endemic to Peninsular India [16].

The genus *Crotalaria* in India, comprises of six sections *Crotalaria* sect. *Hedriocarpae* Wight & Arn., *Crotalaria* sect. *Incanae* (Benth.) Polhill, *Crotalaria* sect. *Calycinae* Wight & Arn., *Crotalaria* sect. *Crotalaria*, *Crotalaria* sect. *Stipulosae* (Baker f.) M.M. le Roux & B.-E.van Wyk and *Crotalaria* sect. *Grandiflorae* (Baker f.) Polhill [6]. Sections *Chrysocalycinae* (Benth.) Baker f. and *Dispermae* Wight & Arnott have been amalgamated with sects. *Hedriocarpae* Wight & Arnott and *Crotalaria* respectively, whilst, sub-sections *Incanae* (Benth.) Bisby & Polhill and *Stipulosae* (Baker f.) Bisby & Polhill have been raised to sectional level [6].

The genus is recognized by a distinctive combination of characters, rostrate keel, inflated pod, hairy style, five plus five anther configuration, paired callosities on the standard petal and the presence of pyrrolizidine alkaloids [6, 15].

During field trips to Western Ghats, we discovered *Crotalaria* plants that did not seem to belong to any of the described species. Critical and detailed comparisons revealed similarities to *Crotalaria albida* Heyne ex Roth, *C*. *epunctata* Dalz., and *C*. *vestita* Baker but several significant differences were also observed.

Here, we describe two new species of *Crotalaria* endemic to Karul Ghat and Panhala region.of Western Ghats (Maharashtra state). We provide for each new species a full taxonomic description, taxonomic comments and photographs, as well as an identification key for all the species of *Crotalaria* from Maharashtra. We also conducted morphometric and phylogenetic analyses to further support the hypothesis.

## Materials and methods

### Ethics statement

All the collecting locations of the new species reported in this study are not in any natural conservation area and no specific permission were required for these locations. The field studies did not involve endangered or protected species. Detailed information (including GPS coordinate) on the location of our study is provided in S1 Appendix, in the Supporting Information, along with the voucher details. Voucher specimens have been deposited in Delhi University Herbarium (DUH) and Botanical Survey of India, Dehradun (BSD).

### Nomenclature

The electronic version of this article in Portable Document Format (PDF) in a work with an ISSN or ISBN will represent a published work according to the International Code of Nomenclature for algae, fungi, and plants, and hence the new names contained in the electronic publication of a PLOS ONE article are effectively published under that Code from the electronic edition alone, so there is no longer any need to provide printed copies. In addition, new names contained in this work have been submitted to IPNI, from where they will be made available to the Global Names Index. The IPNI LSIDs can be resolved and the associated information viewed through any standard web browser by appending the LSID contained in this publication to the prefix http://ipni.org/. The online version of this work is archived and available from the following digital repository: PubMed Central.and LOCKSS.

### Morphological observations

The morphological analysis and description of the two new species are based on the examination of freshly collected and dry vouchers, in addition to flowers preserved in FAA (Formaldehyde-Glacial Acetic Acid-Alcohol). The flowers were rehydrated in water with detergent and dissected to examine the minute details of the corolla under binocular microscope Olympus SZ61. A detailed comparison with the measurements of selected traits and characteristic features of both species are presented in tabular form. Morphological terminology follows Harris and Harris [17] and Hickey and King [18] for vegetative characters, Hewson [19] for indumentum description, and Endress [20] for inflorescence morphology. Both of the new species were found on exposed forest edges, cut slopes, rocky slopes and grasslands. We critically compared the morphology of these specimens with the specimens of *Crotalaria albida*, *C*. *epunctata* and *C*. *vestita* housed in the herbarium at CAL, MH and DUH. We show that these new species differ from their morphologically most similar relatives by measuring various traits on herbarium specimens and personal fresh collections.

The most closely related species were identified based on previous revisionary and systematic works [6–7, 10]. In order to understand the morphological diversity of these species and to ascertain the distinctiveness of both of the new species, a range of specimens, including types as well as voucher specimens were examined from the following herbaria ASSAM, BSD, BSI, CAL, DUH, FRLH, M, MH, SJC, SKU[21]. Specimen images were also studied from the JSTOR Global Plants [22], China Virtual Herbarium [23], Flora of Pakistan [24] and other online herbaria (B, BM, BR, B-WILLD, E, FI, FOB, G-DC, K, L, LINN, NYBG, P, TUB).

To visualize the geographical occurrence of the two new species and the others occurring in the same area, a distribution map was prepared, using a base map from WORLDCLIM [25] and political borders retrieved from Esri Data and Maps [26]. The details of the co-ordinates are presented as S2 Appendix in Supporting Information.

#### Identification of closely related species

A summary of all the diagnostic characters of both the new species and its close allies are presented as tables. Mean trait values and standard error with the minimum and maximum values are provided in Tables 1 and 2.

**Table 1:** Mean trait values and standard error of *Crotalaria suffruticosa*, *C*. *albida* and *C*. *epunctata*. The number of organ observations is n. Minimum and maximum measured values per traits are indicated below each mean.

Trait/Species	*Crotalaria suffruticosa*	*Crotalaria albida*	*Crotalaria epunctata*
Flower Length (mm)	9.8 +/- 0.09 (n = 4) min = 9.5, max = 10	8.56 +/- 0.08 (n = 4) min = 8.3, max = 8.8	10.8 +/- 0.09 (n = 4) min = 10.5, max = 11
Flower width (mm)	6.8 +/- 0.062 (n = 4) min = 6.6, max = 7	4.17 +/- 0.15 (n = 4) min = 3.9, max = 4.7	5.15 +/- 0.10 (n = 4) min = 4.9, max = 5.4
Standard length (mm)	.7 +/- 0.06 (n = 4) min = 8.5, max = 8.8	2.81+/- 0.09 (n = 4) min = 2.56, max = 3.1	10.27+/- 0.21 (n = 4) min = 9.8, max = 10.8
Standard width (mm)	6.6 +/- 0.06(n = 4) min = 6.4, max = 6.8	0.98 +/- 0.06 (n = 4) min = 0.85, max = 1.2	7.32 +/- 0.17 (n = 4) min = 6.8, max = 7.8
Wing length (mm)	77.38 +/- 0.03(n = 4) min = 7.3, max = 7.4	2.2+/- 0.1 (n = 4) min = 1.9, max = 2.5	7.3+/- 0.15 (n = 4) min = 6.9, max = 7.7
Wing width (mm)	2.86 +/- 0.02 (n = 4) min = 2.8, max = 2.9	0.75 +/- 0.02 (n = 4) min = 0.7, max = 0.8	2.4 +/- 015 (n = 4) min = 2, max = 2.8
Keel length (mm)	8.51 +/- 0.01 (n = 4) min = 8.48, max = 8.56	2.85 +/- 0.05 (n = 4) min = 2.7, max = 3	7.95 +/- 0.21 (n = 4) min = 6.9, max = 8
Keel width (mm)	4.005 +/- 0.04 (n = 4) min = 3.89, max = 4.15	1.2 +/- 0.12 (n = 4) min = 0.9, max = 1.5	5.77 +/- 0.09 (n = 4) min = 5.5, max = 6
Calyx tube length (mm)	5.39 +/- 0.01 (n = 4) min = 5.37, max = 5.41	2.63 +/- 0.06 (n = 4) min = 2.45, max = 2.8	8.5 +/- 0.12 (n = 4) min = 8.2, max = 8.9
Gynoecium Length (mm)	2.56 +/- 0.81 (n = 4) min = 2, max = 3.96	1.09 +/- 0.06 (n = 4) min = 0.98, max = 1.3	4.65 +/- 0.13 (n = 4) min = 4.3, max = 5
Gynoecium width (mm)	1.1 +/- 0.17 (n = 4) min = 0.98, max = 1.4	0.42 +/- 0.02 (n = 4) min = 0.35, max = 0.5	0.92 +/- 0.02 (n = 4) min = 0.85, max = 1
Seed length (mm)	1.77 +/- 0.19 (n = 4) min = 1.5, max = 2	2.12 +/- 0.09 (n = 4) min = 1.9, max = 2.4	1.25 +/- 0.09 (n = 4) min = 1, max = 1.5
Seed width (mm)	0.79 +/- 0.12 (n = 4) min = 0.68, max = 1	1.89 +/- 0.03 (n = 4) min = 1.78, max = 2	0.9 +/- 0.03 (n = 4) min = 0.85, max = 1
Leaf length (mm)	39.35 +/- 0.8 (n = 4) min = 38, max = 40	2.27 +/- 0.12 (n = 4) min = 1.9, max = 2.6	0.7 +/- 0.01(n = 4) min = 0.69, max = 0.78
Leaf width (mm)	115.86 +/-0.09 (n = 4) min = 15.75, max = 16	0.91 +/- 0.05 (n = 4) min = 0.78, max = 1.1	0.19 +/- 0.09 (n = 4) min = 0.9, max = 1.4

**Table 2:** Mean trait values and standard error of *Crotalaria multibracteata* and *C*. *vestita*. The number of organ observations is n. Minimum and maximum measured values per traits are indicated below each mean.

Trait/Species	*Crotalaria multibracteata*	*Crotalaria vestita*
Flower length (mm)	7.21 +/- 0.12 (n = 4) min = 6.9, max = 7.5	4.12 +/- 0.13 (n = 4) min = 3.9, max = 4.5
Flower width (mm)	4.18 +/- 0.12 (n = 4) min = 3.8, max = 4.5	6.22 +/- 0.26 (n = 4) min = 5.7, max = 6.9
Standard length (mm)	0.89 +/- 0.0 (n = 4) min = 0.8, max = 1	8.57 +/- 0.23 (n = 4) min = 8, max = 9
Standard width (mm)	0.95 +/- 0.02(n = 4) min = 0.8, max = 1	5.2 +/- 0.15 (n = 4) min = 4.9, max = 5.5
Wing length (mm)	0.94 +/- 0.022 (n = 4) min = 0.8, max = 1	7 +/- 0.28 (n = 4) min = 6.5, max = 7.8
Wing width (mm)	0.38+/- 0. 01(n = 4) min = 0.3, max = 0.4	2.1 +/- 0.11 (n = 4) min = 1.9, max = 2.4
Keel length (mm)	0.94 +/- 0.03 (n = 4) min = 0.8, max = 1	6.67 +/- 0.26 (n = 4) min = 6, max = 7.2
Keel width (mm)	0.38 +/- 0.008 (n = 4) min = 0.3, max = 0.4	6 +/- 0.26 (n = 4) min = 5.4, max = 6.7
Calyx tube length (mm)	0.95 +/- 0.02 (n = 4) min = 0.8, max = 1	8.15 +/- 0.23 (n = 4) min = 7.6, max = 8.7
Gynoecium length (mm)	0.27+/- 0.01 (n = 4) min = 0.2, max = 0.3	3.15 +/- 0.28 (n = 4) min = 2.6, max = 3.9
Gynoecium width (mm)	0.36+/- 0.02 (n = 4) min = 0.3, max = 0.4	1.15 +/- 0.13 (n = 4) min = 0.9, max = 1.5
Seed length (mm)	0.16 +/- 0.01 (n = 4) min = 0.1, max = 0.2	1.18 +/- 0.09 (n = 4) min = 1, max = 1.4
Seed width (mm)	0.056 +/- 0.002 (n = 4) min = 0.05, max = 0.06	0.55 +/- 0.144 (n = 4) min = 0.2, max = 0.9
Leaf length (mm)	14.865 +/- 0.053 (n = 4) min = 14.76, max = 15	36.97 +/- 1.28 (n = 4) min = 34, max = 39
Leaf width (mm)	6.885 +/-0.044 (n = 4) min = 6.7, max = 7	17.95 +/- 0.55 (n = 4) min = 16.8, max = 19.2

### Taxa sampling

The field surveys and plant collection trips were conducted in 2011 and 2015 in Kolhapur, Maharashtra. Voucher specimens are deposited in BSD (Botanical Survey of India, Dehradun, India) and DUH (Delhi University Herbarium, India). Of the total of 37 species occurring in Maharashtra, we collected 33 (92%). Of these 33 species collected, 40% are endemic. A total of 94 accessions for the ITS marker and 86 for the plastid marker *matK* (including outgroups) of which 72 accessions represent Indian species of *Crotalaria* [8, 15]. *Bolusia amboensis* (Schinz) Harms and *Euchlora hirsuta* (Thunb.) Druce were included as outgroup for the analyses following the molecular study of Boatwright et al. [3]. Voucher details along with author citations and the GenBank accession numbers have been provided in the additional information S1 Appendix. ITS and *matK* sequences of outgroup taxa and the African *Crotalaria* species were retrieved from GenBank.

### Molecular methods

Genomic DNA was extracted using a DNeasy plant mini kit (Qiagen, Amsterdam, The Netherlands). DNA amplification and sequencing of the ITS region was performed using the primers ITS 1 and ITS 2 [27]. The polymerase chain reaction (PCR) for the ITS region was performed with standard methods [8]. The *matK* region was amplified and sequenced as one segment using Barcoding primers of Jing-Yu et al. [28]. Reaction conditions for the *matK* region include denaturation at 94°C for 3 min followed by 35 cycles of 1 min at 94°C, 1 min at 52°C and 1min at 72°C, followed by a final extension at 72°C for 5 min in an applied biosystems thermal cycler. PCR products were checked for the presence of appropriate bands on a 0.8% agarose gel, purified, and sequenced at SciGenome; Kochi Kerala, India. Sequences comprised of ITS1, 5.8S and ITS2 regions and *matK* s. For the *matK* region, forward and reverse sequence reads were using DNA Baser v.4.36 [29]. Consensus sequences for all accessions were imported into Clustal X [30] and MAFFT v.7 [31–32] in which the sequences were aligned followed by manual adjustments in Mesquite v.2.72 [33]. Gaps were treated as missing data. For the ITS region, chromatograms were using Sequencher (Gene Code corporation, USA) [34] and partial bases were converted to N’s. A total of 105 nucleotide sequences (including all outgroups) for ITS and 113 nucleotide sequences (including all outgroups) for *matK* were. 98 sequences representing Indian accessions have been deposited in GenBank (S1 Appendix).

#### Phylogenetic analyses

**Fig 1:**
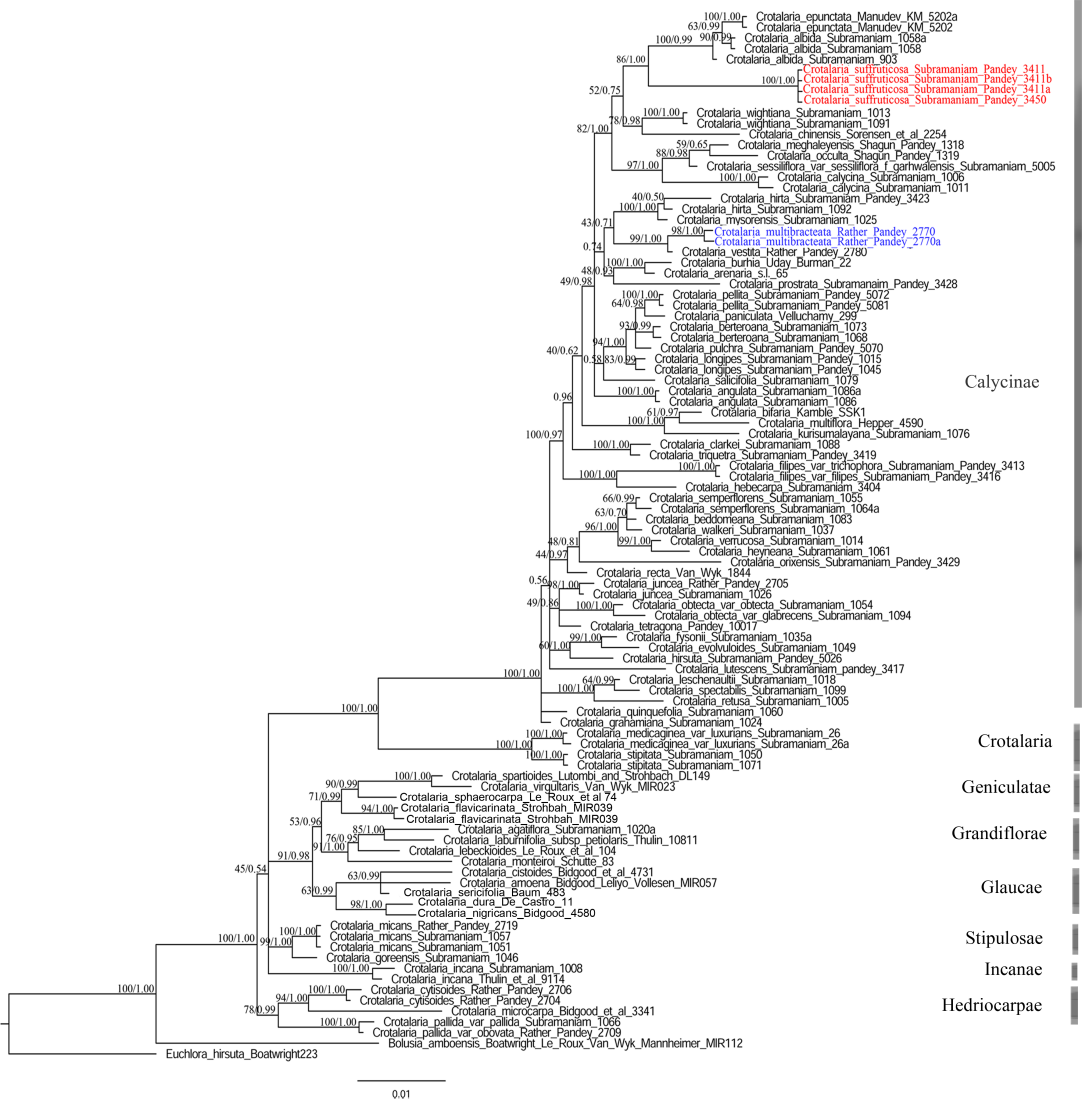
Maximum likelihood tree of the genus *Crotalaria*, constructed using RAxML, with bootstrap support values and Bayesian posterior probabilities indicated above the branches. Since Bayesian analyses resulted in almost the same topology, only the tree constructed from RAxML has been presented here. The new species are colored in red and blue.

Independent phylogenetic analyses were conducted for ITS and *matK* regions. Both regions were concatenated using Mesquite v.2.72 [33]. The latter is a part of the non-recombining plastid genome and are frequently combined for phylogenetic reconstruction [35–37]. For the purpose of this study both regions were combined for analyses. No major conflicts (incongruence) were identified between single-region analyses, which showed broad similar phylogenetic groups. The best-fitting model of nucleotide substitution was selected using the Akaike information criterion [38] and implemented in the program jmodelTest 0.1.1 [39–40]. It was found to be GTR+G, with the lowest AIC score and highest log-likelihood score for both the regions. Bayesian analysis was performed using MrBayes 3.1.2 [41]. Parameters for the evolutionary model were set to default and the state frequency parameter for stationary nucleotide frequency of the rate matrix was fixed. The number of chains was set to four with three heated and one cold chain. Two runs were executed in parallel. Analyses were run for 7,000,000 generations until stationarity (standard deviation below 0.01). In each run, trees were sampled every 100 generations with a sample frequency of 10. The parameters were summarized after excluding 25% of the samples (burn-in) based on the inspection of log-likelihoods of sampled trees after stationarity was reached. The Potential Scale Reduction Factor (a convergence diagnostic) approached 1.0 for all the parameters suggesting good sampling from the posterior probability distribution with no spread. Trees were summarized by the sumt burnin command yielding a cladogram showing posterior probabilities and clade credibility for each split and a phylogram with mean branch lengths (Fig 1). Maximum likelihood (ML) analyses were performed using RaxML v.1.3 [42]. The heuristics of RAxML-III belong to the class of algorithms, which optimize the likelihood of a starting tree already comprising all sequences. In contrast to other programs, RAxML-III starts by building an initial parsimony tree. For likelihood (ML) analyses, settings were ‘‘ML+ thorough bootstrap” with 100 (replicate) runs and 1000 (bootstrap) repetitions with the GTR+G model (six general time-reversible substitution rates, assuming gamma rate heterogeneity).

### Trait measurements and statistical analyses

**Fig 2:**
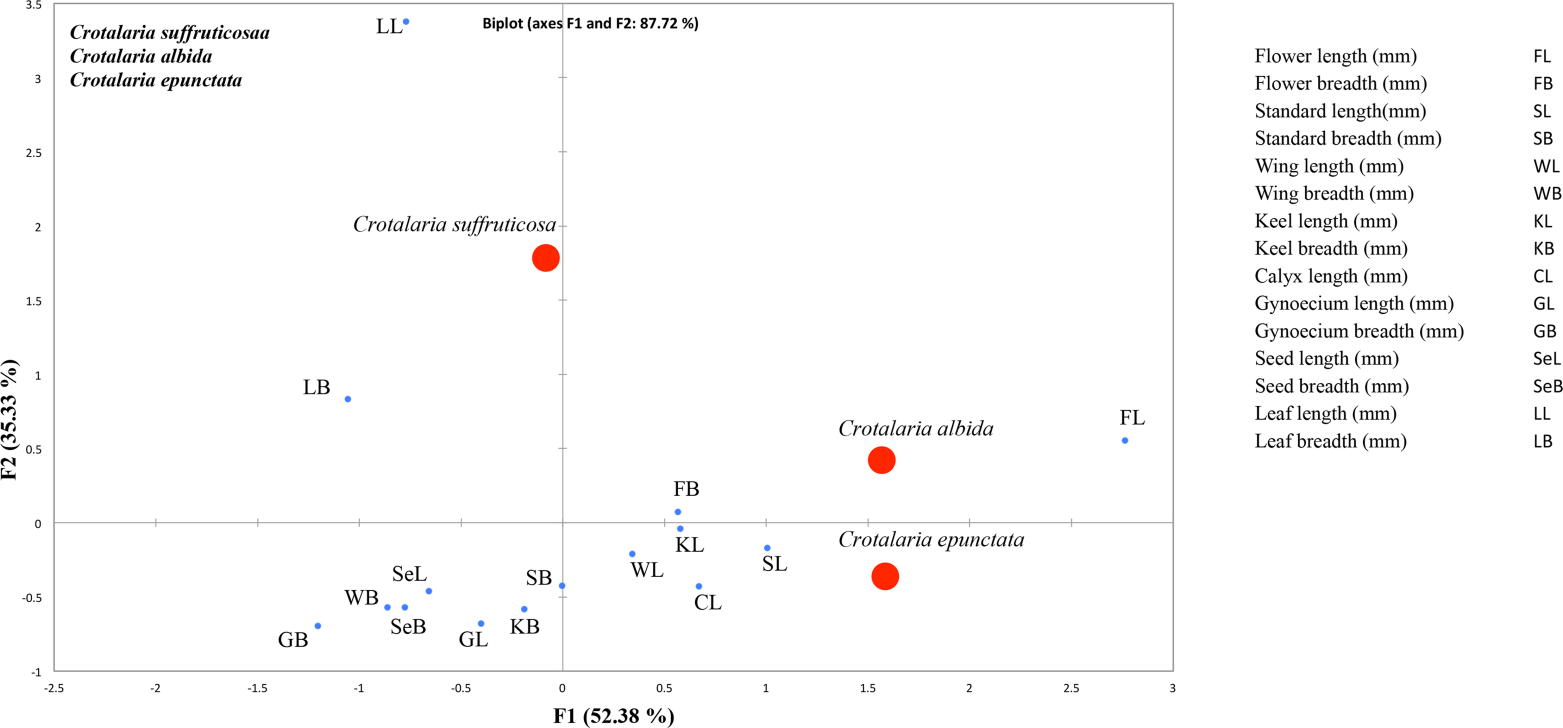
Principal component analysis on morphological traits comparing the new species *Crotalaria suffruticosa* and morphologically most similar species *C*. *albida* and *C*. *epunctata*.

**Fig 3:**
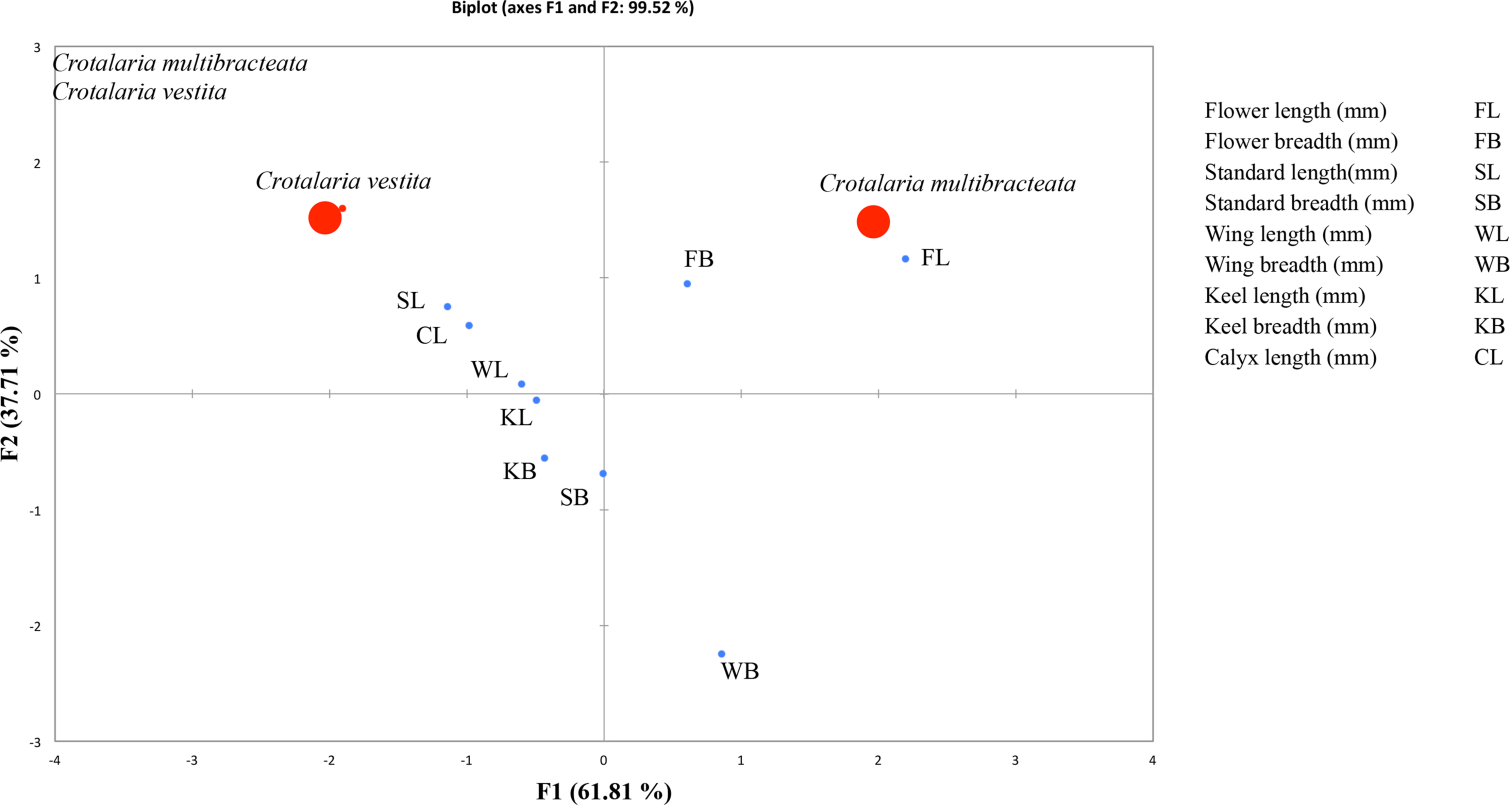
Principal component analysis on morphological traits comparing the new species *Crotalaria multibracteata* and morphologically most similar species *C*. *vestita*.

To evaluate whether the two new species differed from the presumably closest relatives by understanding which traits were most relevant with regard to their identification, we performed a Principal Component Analysis (PCA), using Microsoft R Excel 2000 XLSTATC-Pro v.7.2 (Addinsoft, Inc., Brooklyn, New York) [43], and BioDiversity Pro v.2 [44] where the significance level was set at 5% (Figs 2 and 3). PCA is one of the many ways to analyse the structure of a given correlation matrix. The Principal Component Analysis method (PCA) may be useful in selecting from among the great number of morphometric characters, especially those that have some taxonomical value. Such a necessity is occurring within genera which species are very uniform in morphological structure and there are weak qualitative characters differentiating them [45]. The specimens observed for this study are listed in the section below as “specimens examined”. The following traits were measured for each of the five species: *Crotalaria albida*-*C*. *epunctata*-*C*. *suffruticosa* and *Crotalaria multibracteata-C*. *vestita* flower length, flower width, standard length, standard width, wing length, wing width, keel length, keel width, calyx tube length, gynoecium length, gynoecium width, seed length, seed width, leaf length and leaf width. For each specimen, the mean values of the above traits were calculated (for example, mean corolla length of the three flowers of an individual). These means were then used to calculate the significant ratios.

## Results and discussion

### Phylogenetic analyses

The DNA sequencing of the nuclear ITS region of the two new species generated a sequence length of 792 bp in *Crotalaria multibracteata* (GenBank numbers:KY321450, KY321451) and 790 bp in *C*. *suffruticosa* (GenBank numbers: KY321453, KY321454, KY321455, KY321456) and the *matK* region of the two new species generated a sequence with 784 bp in *C*. *multibracteata* and 782 bp in *C*. *suffruticosa*. The concatenation of both the sequences of the new species resulted in 1576 and 1572 bp sequence of *Crotalaria multibracteata* and *C*. *suffruticosa* respectively in the aligned matrix (without gaps). The complete aligned matrix comprised of 98 accessions containing 1660 characters.

The phylogeny constructed under Maximum likelihood and Bayesian approach revealed broadly the same topology (Fig 1). The tree resolves into eight major clades representing eight sections out of the eleven sections as proposed by Le Roux et al. [6] for the genus *Crotalaria*. The eight clades corresponds to the following sections (as marked in the tree) viz., Calycinae, Crotalaria, Geniculatae, Grandifloirae, Glaucae, Stipulosae, Incanae, Hedriocarpae. All these major clades are well supported (clades for which the parsimony and likelihood support values are more than 80 BS and Bayesian posterior probability values are more than 0.9 pp), and are congruent with earlier phylogenetic analyses [8]. The phylogeny supports the status of the genus as monophyletic (100 BS/1.00 pp). Most of the Indian species (51 species in Calycinae clade) of *Crotalaria* forms a part of the *Calycinae* clade (100 BS/1.00 pp) which is congruent with the earlier phylogenetic work of Subramaniam et al. [8]. India hosts the maximum number species in the *Calycinae* clade which is mainly characterized by its simple leaved species (exception: *C*. *orixensis*). Within this simple leaved clade, *Crotalaria suffruticosa* forms a distinct sub-clade (100 BS/1.00 pp) with *C*. *albida* and *C*. *epunctata* (86 BS/1.00 pp) in the *Calycinae* clade. The latter two species are strongly supported as sister to one another (100 BS/0.99 pp). *Crotalaria multibracteata* is sister to *C*. *vestita* (99 BS/1.00 pp), together forming a clade which is sister to *C*. *hirta* and *C*. *mysorensis* albeit with low support. (0.71 pp).

The phylogeny demonstrates the distinct status of the new species *C*. *suffruticosa* and *C*. *multibracteata*, and resolves their position in separate subclades within the Calycinae clade (Fig 1). The new species *Crotalaria suffruticosa* differs from *C*. *albida* and *C*. *epunctata* in 16 nucleotide substitutions, and one inversion at site 520 respectively (in both regions). It also differs from *Crotalaria albida* and *C*. *epunctata* in two insertions of length two and 12 at sites 520–521 and 895–906 respectively. *Crotalaria suffruticosa* shares similarity with *C*. *albida* and *C*. *epunctata* at sites 740 and 976 with two substitutions. *Crotalaria multibracteata* differs from *C*. *vestita* in five substitutions and one inversion at site 1564. It is similar to *C*. *vestita* in having eight substitutions and one insertion at site 264–265 respectively.

### Morphometric analyses

Principal Component Analysis in the form of Pearson’s co-efficient showed the significant characters which help in morphological differentiation between the new species and the species most similar in gross morphology (Figs 2 and 3; Tables 1 and 2). This morphometric analyses have been proved to be very useful in showing correlations between the variables or distance among groups and in estimating the contribution of each character. The significant characteristic ratios, which contributed to the uniqueness of the new species, are indicated in the Figs 2 and 3. The mean diagnostic sizes concentrated the new species from their close allies into different groups. Traits indicated close to the respective species, in the PCA plot are governed by that species to the maximum.

### Taxonomic treatment

This addition of the new species in Western Ghats envisages an addition to the existing 36 species in the hotspot region of Indian sub-continent. Our group is investigating the biogeography of the genus, which in future will contribute to the understanding of the present-day distribution and floristic exchanges in the Western Ghats region. This will in future contribute to our understanding of the causes of disjunct distribution of the species of *Crotalaria* in India.

**Fig 4:**
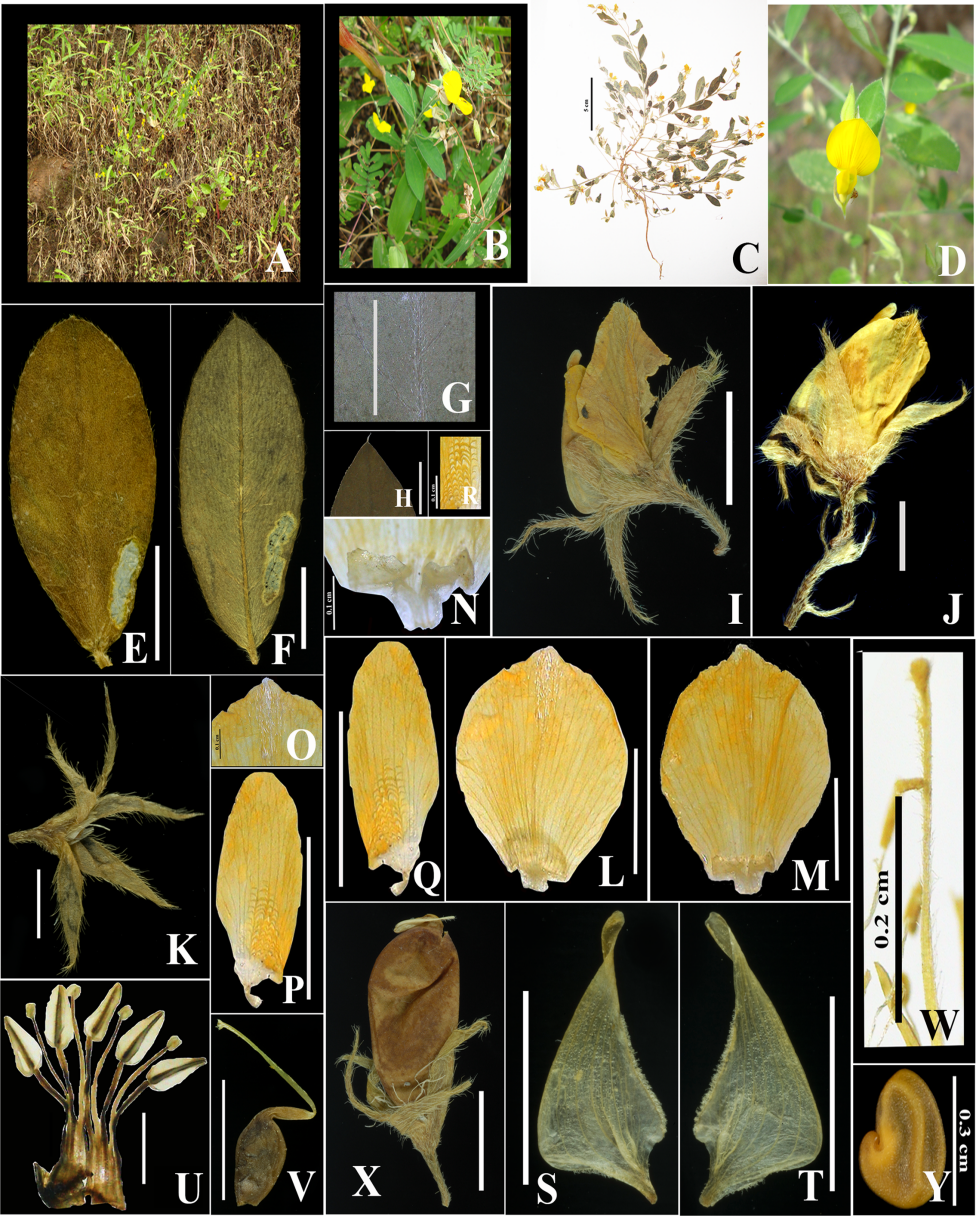
A–Y. *Crotalaria suffruticosa*. A. Habit. B. Plant twig showing leaves and flowers. C. Herbarium specimen of the new species. D. Close-up of the flower in field. E. Adaxial leaf surface with white sparse pubescent vestiture. F. Abaxial surface with pubescent vestiture. G. Close-up of abaxial leaf surface showing prominent hairs on the midrib region. H. Close-up of mucronulate leaf apex. I. Flower showing calyx, corolla and pedicel. J. Position of bract (base of pedicel) and bracteoles (middle of pedicel). K. Bi-lipped calyx with pubescent surface. L. Adaxial surface of standard. M. Abaxial surface of standard. N. Planar callosities. O. Close-up of silky pubescence on standard dorsal apex. P-Q. Wing petals. R. Close-up of cavae. S-T. Keel petals, angled with lower third curvature and ciliate glabrous vestiture. U. Anthers in 5 + 5 arrangement (one missing), with five carinal/basifixed/sagittate anthers and five small dorsifixed ovoid anthers. V. Gynoecium showing ovary, style and stigma. W. Close-up of style showing trichomes in two parallel rows(parallel) and brush type stigma. X. Fruit showing glabrous surface and prominent beak. Y. Cordiform seed, golden brown color. Scale bar 0.5 cm unless indicated otherwise.

***Crotalaria suffruticosa*** S. Subramaniam & A.K. Pandey, sp. nov.–(Fig 4)

[*urn*:*lsid*:*ipni*.*org*:*names*:*XXXXXXX]*

#### Type

INDIA. Maharashtra: Kolhapur district, Vaibhavwadi taluka, Karul Ghat, road side, 960 m, Lat. 16° 31’ 58” N, Long. 73° 49’ 5” E, 12 October 2011, flowering and fruiting, *S*. *Subramaniam & A*. *K*. *Pandey 3450* (holotype: DUH!; isotypes: DUH!, BSD!).

Ascending or mainly erect suffruticose, branched herb, up to 0.5 m high, slightly woody from near the base. Stems terete, branches tomentose with white trichomes prominent on the younger branches. Leaves simple, alternate; petiole up to 0.1 cm long; lamina elliptic to oblanceolate, ca. 4.0 × 1.6 cm, base acute, apex acute or mucronulate, margins entire and ciliate, venation pinnate-brochidodromous, with white velutinous trichomes beneath, glabrescent above, exstipulate. Inflorescence a terminal raceme; peduncles up to 8 cm long bearing up to 7 flowers, axillary peduncles up to 4 cm long, with one to three flowers. Flowers ca. 1.0 × 0.7 cm across; bracts membranous, linear lanceolate, up to 3.0 mm long, with white silky pubescence; pedicels 0.2–0.3 cm long reflexed downwards; bracteole single, inserted on the pedicel, linear pubescent, up to 3 mm long, with silky pubescence and slightly involute margin. Calyx 5–lobed, bi-lipped, upper lip consisting of two sepals and lower lip, three sepals each, ca. 5.45 mm long, connate at base, tapering to apex, apex acute, hirsute; tube ca. 1.63 mm long, margins ciliate and slightly involute. Corolla yellow, exserted from the calyx; vexillum obovate-elliptic, ca. 8.90 × 6.90 mm, claw ca. 0.87 mm long, with paired planar callosities of ca. 1.14 × 0.52 mm at the base; trichomes ca. 0.45 mm long, on almost entire midvein and spreading towards upper portion of the petal; wing petal ca. 7.48 × 2.89 mm, multi-veined, claw ca. 0.9 mm long, cavae (sculpturing/ridges) 0.19–0.41 mm long, on the lower middle of the wing; keel angled, curvature on lower third, alae absent, claw ca. 0.91 mm long, glabrous, except the upper edge ciliate, ca. 8.56 × 4.15 mm, upper portion tapering, rostrate, beak twisted. Staminal sheath ca 0.91 mm long; free filaments 1.3–4.5 mm long; anthers dimorphic, basifixed, longer, ensiform, ca. 1.31 mm long, dorsifixed, shorter, orbicular, ca. 0.27 mm long, filaments glabrous. Ovary sessile, linear, ca. 2.18 mm long, inflated, style ca. 4.1 mm long, sub-geniculate, covered with trichomes in two lines; stigma brush type and contracted, ca. 0.31 mm long, covered with trichomes; ovules up to 5 per fruit. Pods brown, oblanceolate, ca.1.4 × 0.6 cm, exserted (0.6 cm long), glabrous, with short beak and persistent style. Seeds yellow to light brown matte, 1.5–2.0 × 0.68–1.0 mm.

#### Phenology

Flowering September to December, fruiting November to February.

#### Distribution, habitat and ecology

**Fig 5:**
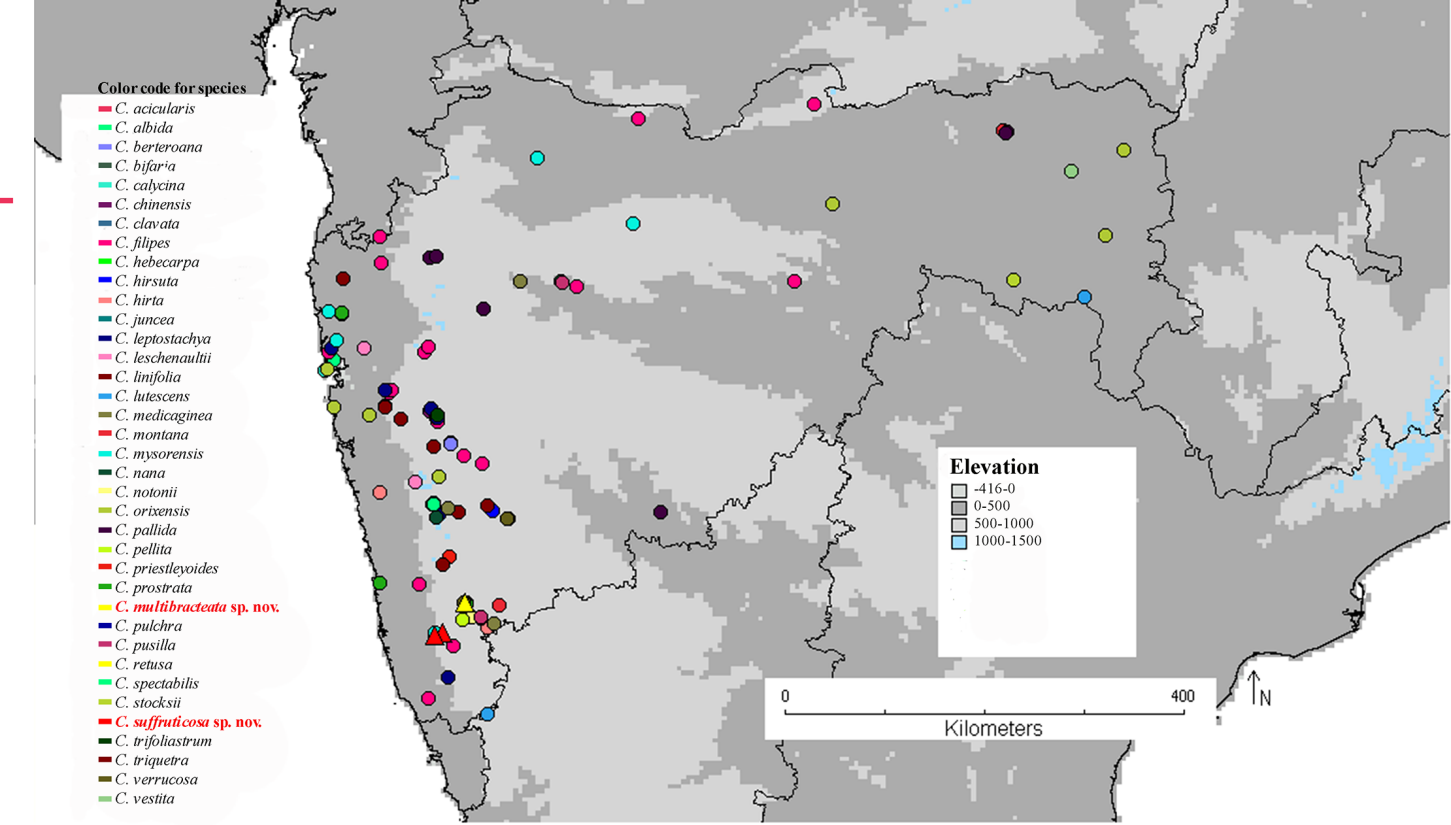
Distribution map of all 37 *Crotalaria* species found in Maharashtra including the two new species *Crotalaria suffruticosa* and *C*. *multibracteata*. The color and symbols specify the distribution of different species and the triangle (red: *C*. *suffruticosa*, yellow: *C*. *multibracteata*) designates the distribution of the new species.

*Crotalaria suffruticosa* grows on cut slopes, exposed forest edges and rocky slopes of the Karul Ghat region (Fig 5). Karul Ghat is a stretch of typical grassland and forest edges. The temperature has a relatively narrow range between 10°C to 35°C. Mean relative humidity in summer (March-May) is up to 65%, it is 87% during wet weather (June–October) and 63% in winters (November–February) [46].

*Crotalaria suffruticosa* has a sympatric distribution with *C*. *albida*. Other plants associated with *C*. *suffruticosa* include *Tephrosia tinctoria* (L.) Pers., *Terminalia chebula* Retz., *Exacum tetragonum* Roxb. along with other grass species.

#### Etymology

The species is named for its suffruticose habit.

#### IUCN conservation status

Endangered (EN). The species is known only from two sites, the type locality and another adjacent area near the type locality. In accordance with two of the IUCN [47–48] criteria, it should be best considered endangered, according to the preliminary investigations made because it meets criterion of points A-E of section V of IUCN.

#### Species recognition

*Crotalaria suffruticosa* resembles its most closely related species *C*. *albida* and *C*. *epunctata* in having a pubescent stem surface, inflorescence being a terminal/axillary raceme, yellow corolla, bilipped calyx with pubescent surface and ciliate margin, bracts and bracteoles with white silky pubescence, ovate-elliptic-oblong standard, gynoecium surface glabrous, brush type stigma, pod elliptic oblong with glabrous surface. It differs from *C*. *albida* and *C*. *epunctata* in habit, height, leaf size and margin, inflorescence length and number of flowers/inflorescence, bracteole position, standard apex, callosity type, keel shape, curvature and vestiture, style type and trichomes details of which are summarized below and in Table 3. *Crotalaria suffruticosa* has a stiff erect, suffruticose habit, up to 50 cm high (vs. up to 80 cm in *C*. *albida* and up to 1 m in *C*. *epunctata*), leaves up to 4 cm long with simple margin (vs. up to 5 cm in *C*. *albida* and up to 10 cm in *C*. *epunctata*, both with non-ciliate margins), inflorescence up to 8 cm in length (vs. up to 15 cm in *C*. *albida* and up to 28 cm in *C*. *epunctata*) and up to 7 flowers/ inflorescence (vs. up to 26 flowers/inflorescence in *C*. *albida* and up to 20 flowers/inflorescence in *C*. *epunctata*), bracteoles present on the middle of the pedicel (vs. attached at the base of calyx in *C*. *albida* and *C*. *epunctata*), notched standard apex (vs. rounded standard apex in *C*. *albida* and *C*. *epunctata*), planar callosity (vs. ridge callosities in *C*. *albida* and *C*. *epunctata*), keel angled (vs. keel sub-angled in *C*. *albida* and *C*. *epunctata*), with lower third curvature (vs. below the middle in *C*. *albida* and *C*. *epunctata*) and ciliate glabrous vestiture (vs. lanate vestiture in *C*. *albida* and *C*. *epunctata*), style with hairs arranged in two rows (vs. hairs in one row) and subgeniculate (vs. geniculate). A comparative morphology of all the characters between *Crotalaria suffruticosa* and its close allies has been provided in Table 3. Based on the morphological and molecular evidences, the plant collected from Karul Ghat, Kolhapur, is best placed in *Crotalaria* sect. *Calycinae* based on keel curvature, twisted beak, calyx more than half as long as the keel to longer than the keel, often bilipped and leaves usually simple.

**Table 3:** Diagnostic features and comparative morphology of *Crotalaria suffruticosa* with its morphologically most similar species (based on Sanjappa 1991 [49], Ansari 2008 [10], Le Roux et al. 2013 [6], Subramaniam et al. 2013, 2015 [8, 15]; present study. Bold font represents the main distinguishing features of the new species with its close allies.

Characters	*Crotalaria suffruticosa*	*Crotalaria albida*	*Crotalaria epunctata*
**Habit**	**Stiff, ascending or erect herbs**	**Slender, Erect or procumbent herb**	**Slender subshrub**
**Height**	**Up to 50 cm**	**Up to 80 cm**	**Up to 100 cm**
Stem surface	Pubescent with white hirsute	Adpressed brown pilose-pubescent	Adpressed pubescent, older less hairy
**Leaf size**	**Up to 4 cm long**	**Up to 4.9 cm long**	**Up to 10 cm long**
Petiole	Up to 0.1 cm long	0.07–0.15 cm long	0.1–0.2 cm long
Petiole surface	White silky pubescent	Pilose	Pubescent
Leaf shape	Elliptic to oblanceolate	Linear, oblong-lanceolate or oblanceolate	Obovate or obovate-oblong
Leaf apex	Acute, mucronulate	Retuse, sub-acute or obtuse, mucronulate	Obtuse, slightly retuse or sub-truncate
Leaf surface	Upper surface glabrescent with white hairs, lower surface pubescent, non pellucid dotted	Upper surface glabrous or pilose, lower surface pilose-tomentose, pellucid dotted	Upper surface glabrescent, lower surface slightly paler and pilose pubescent, non pellucid dotted
**Leaf margin**	**Simple and ciliate**	**Slightly involute and non ciliate**	**Slightly involute and non ciliate**
Inflorescence	Terminal or axillary raceme	Terminal or rarely axillary raceme	Terminal raceme (rarely axillary) or panicle
**Inflorescence length**	**Up to 8 cm long, axillary up to 4 cm**	**Up to 15 cm long**	**Up to 28 cm long**
**Number of flowers per inflorescence**	**Up to 7, axillary raceme with 1 or few flowers**	**Up to 26**	**Up to 20**
Bracts on peduncle	1	1	Extra bracts on peduncle (more than 4)
Flower color	Yellow	Pale yellow or whitish	White or yellow
Flower size	1.0 x 0.7 cm	0.7–1 x 0.4 cm	0.6–1.2 x 0.4–0.6 cm
Calyx length	ca. 0.54 cm	0.7–1.2 x 0.15–0.3 cm	0.7–0.9 x 0.1–0.3 cm, ca. 0.1cm
Calyx surface	White hairs	Sericeous hairs	Golden brown hairs
Calyx margin	Ciliate and slightly involute	Densely ciliate	Ciliate
Calyx apex	Acute	Sub-acuminate	Acute
Calyx shape	Connate at base tapering above	Oblong-lanceolate	Oblong-lanceolate
Bract size	0.3 cm long	0.15–0.2 cm long	0.1–0.15 cm long
Bract surface	White silky pubescent	Pilose	Pubescent
Bract margin	Slightly involute	Entire	Entire
Bract shape	Linear lanceolate	Setaceous, minute	Linear
Bracteole shape	Single, linear pubescent	Linear	Linear
Bracteole surface	Pubescent	Pilose	Pubescent
Bracteole size	0.3 cm long	0.1–0.2 cm long	0.2–0.4 cm long
Bracteole margin	Slightly involute	Simple	Simple
**Bracteole position**	**On the middle of the pedicel**	**Attached to the base of calyx**	**Below the calyx**
Bracteole number	One	Two	Two
Standard shape	Obovate-elliptic	Obovate-oblong or sub-orbicular	Obovate-oblong or sub-orbicular
Standard dorsal surface	Apex and mid-rib silky pubescent	Pilose on the back at the apex	Pilose at the middle or up to the base
Standard size	ca. 0.86 × 0. 68 cm	0.7–0.9 × 0.27 cm	0.6–1.1 × 0.4–0.85 cm
Standard color	Yellow	Pale yellow or whitish	White or yellow
**Standard apex**	**Notched**	**Rounded**	**Rounded**
**Callosite**	**Planar**	**Ridge**	**Ridge**
Wing size	ca. 0.74 × 0. 28 cm	0.5 × 0.1–0.2 cm	0.6–0.8 × 0.2–0.3 cm
Claw size	ca. 0.09 cm	0.06–0.15 cm long	ca. 0.1 cm
Keel size	ca.0.85 × 0. 41 cm	0.55–0.65 × 0.25–0.6 cm	0.5–0.9 × 0.45–0.6 cm
**Keel shape**	**Angled**	**Subangled**	**Subangled**
Keel alae	Absent	Present	Present
**Keel curvature**	**Lower third**	**Below the middle**	**Below the middle**
**Keel vestiture**	**Ciliate glabrous**	**Lanate**	**Lanate**
Keel beak	Twisted up to 90°	Spirally twisted up to 90°	Spirally twisted up to 90°
Androecium size	Staminal sheath ca. 0.09 cm long	Staminal sheath 0.18–0.25 cm long	Staminal sheath 0.2–0.3 cm long
	Filaments 0.13–0.45 cm long	Filaments 0.13–0.35 cm long	Filaments 0.2–0.45 cm long
	Longer anther (oblong or linear) ca. 0.131 cm long	Longer anther (oblong or linear) 0.13–0.17 cm long	Longer anther (linear-oblong) 0.1–0.17 cm
	Shorter anther (ovoid) ca. 0.027 cm long	Shorter anther (ovoid) 0.02–0.05 cm long	Shorter anther (ovoid) 0.03–0.04 cm
Gynoecium size	ca. 0.21 cm long	0.45 × 0.15–0.2 cm	0.4–0.5 × 0.1–0.15 cm
**Style hairs**	**Two rows**	**One row**	**One row**
Style length	ca. 0.41 cm long	0.45–0.57 cm long	0.3–0.4 cm long
**Style bent from ovary/curved**	**Subgeniculate**	**Geniculate**	**Geniculate**
Pod stalk	ca. 1 cm	Sub-sessile	ca. 0.05 cm long
Pod shape	Elliptic-oblong	Obovate-oblong	Oblong or obovate-oblong
Pod size	ca.1.4 × 0.6 cm	1.0–1.3 × 0.4–0.5 cm	0.7–1.1 × 0.2–0.4 cm
Number of seeds per pod	-	8 to 9	6 to 8 (12)
Seed size	0.17–0.2 × 0.068–0.1 cm	0.25 × 0.2 cm long	0.1–0.15 × ca. 0.1 cm
Seed color	Golden brown	Brown or brownish black	Brown
Seed surface	Smooth, glossy	Smooth, glossy	Smooth, glossy

#### Additional specimen examined

TYPE: INDIA. Maharashtra: Kolhapur district, Vaibhavwadi taluka, Karul Ghat, road side, 216 m, Lat. 16° 30’ 42” N, Long. 73° 49’ 34” E, 12 October 2011, flowering and fruiting, *S*. *Subramaniam & A*. *K*. *Pandey 3411* (paratype: DUH!).

**Fig 6:**
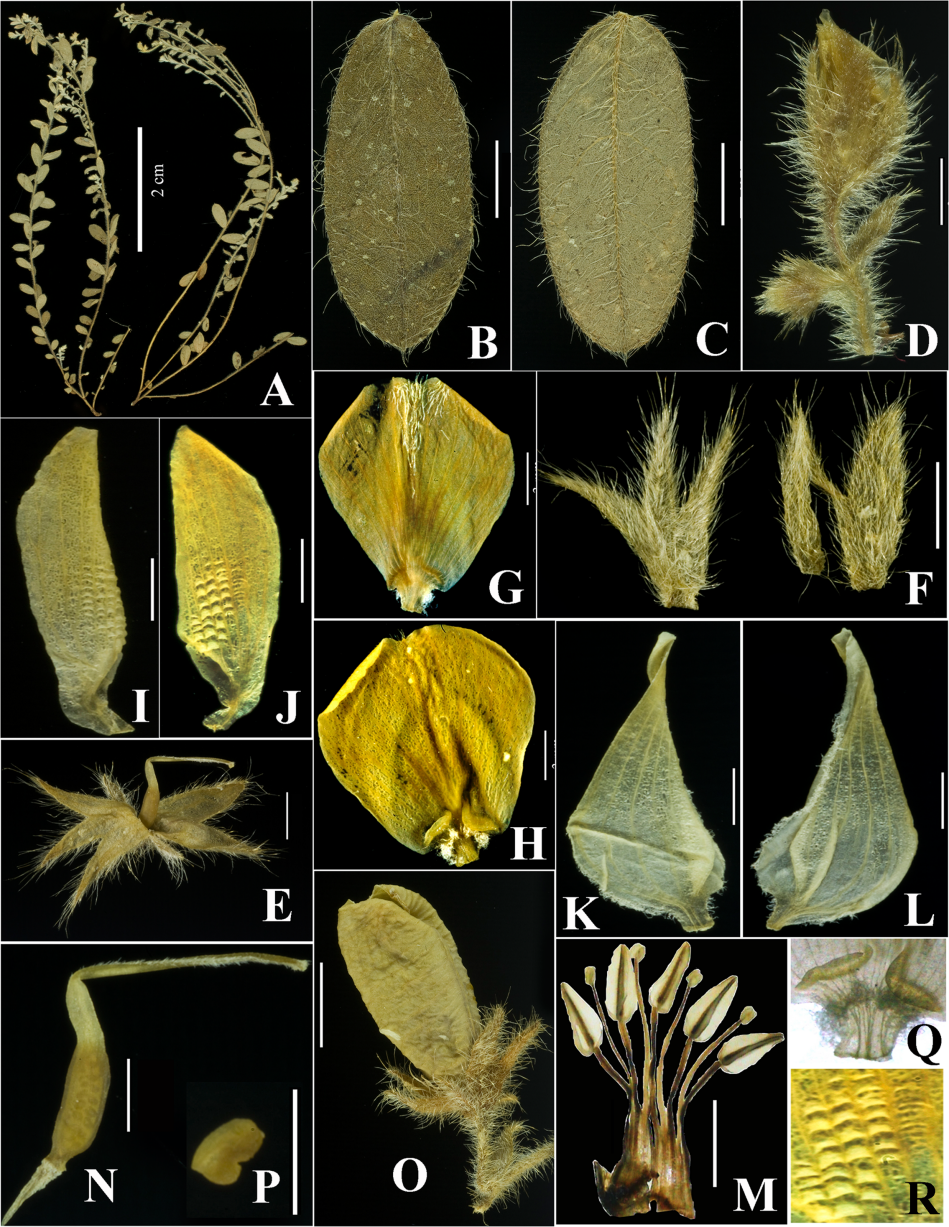
A–R. *Crotalaria multibracteata*. A. Plant twig showing leaves and flowers. B-C. Adaxial and abaxial leaf surface with hirsute vestiture and ciliate margin. D. Flower showing pedicel, bracteoles and calyx with corolla inserted (all three with dense pubescence). E. Bilipped calyx with pubescent-densely ciliate surface. F. Both lips of calyx dissected show surface and bi-lipped condition. G. Adaxial surface of standard with pubescent apex. H. Abaxial surface of standard. I-J. Wing petals. K-L. Keel petals, sub-angled with below the middle curvature and lanate vestiture. M. Anthers in 5 + 5 arrangement (one missing), with five carinal/basifixed/sagittate anthers and five small dorsifixed ovoid anthers with their filaments fused to form a staminal sheath. N. Gynoecium showing ovary, style and stigma. O. Fruit showing glabrous surface, and densely pubescent calyx. P. Seed reniform, golden brown with smooth surface. Q. Close-up of lameliform callosities. R. Close-up of cavae.

***Crotalaria multibracteata*** S.A. Rather & A.K. Pandey, sp. nov.–(Fig 6)

[*urn*:*lsid*:*ipni*.*org*:*names*:*XXXXXXX]*

#### Type

INDIA. Maharashtra: Kolhapur district, Panhala, 875 m, Lat. 16° 49' 7.4562" N, Long. 74° 6' 28.5336" E, 25 December 2015, *S*. *A*. *Rather & A*. *K*. *Pandey 2770* (holotype: DUH!; isotypes: DUH!, BSD!).

Procumbent slender herb much branched, up to 0.50 m high. Stems with—prominent velutinous white trichomes. Leaves simple, alternate, sessile, ovate to oblong, 1.0–1.5 × 0.4–0.7 cm, obtuse at base, abruptly acute at apex, margins simple, with hirsute trichomes on both surfaces; exstipulate. Inflorescence terminal or leaf opposed; terminal peduncles up to 2 cm long bearing up to 2–4 flowers. Flowers 6.0–7.5 × 3.5–4.5 mm across; multiple bracts on peduncle (more than 4), linear, up to ca. 1 mm long, with densely silky pubescent hairs and ciliate margins; bracteole single, at the middle of the pedicel, densely silky pubescent. Calyx 5-lobed, bi-lipped, upper lip with two sepals and lower lip with three sepals, ca. 0.7–1 mm long, paleaceous, margins densely ciliate. Corolla light yellow; vexillum obovate-orbicular, 0.8–1.0 × 0.6–1.0 mm with paired lamelliform callosities present on blade only, emarginate apex, claw ca. 1.0× 0.24 mm at the base; trichomes ca. 0.21 mm long, on apex and spreading up to half of midvein; wing petal ca. 0.3–1.0 × 0.1–0.4 cm, multi-veined, claw ca. 0.06–0.08 × 0.04–0.05 mm long, cavae (sculpturing/ridges) 0.18–0.35 mm long, on the lower middle on the external portion of the petals and on the upper side of the petal; keel petals sub-angled, below the middle, alae absent, vestiture lanate vestiture, ca. 0.8–1.0 × 0.3–0.4 mm, upper portion tapering, rostrate, tip twisted up to 90°, beak ca. 1.0 mm long, claw ca. 0.56 mm long, margins lanate. Staminal sheath ca.0.2–0.5 mm long; free filaments 1.3–1.7 mm long; anthers dimorphic, basifixed longer anthers dithecous, oblong or linear, ca. 0.1–0.9 mm long, dorsifixed shorter anthers ovoid, ca. 0.2–0.4 mm long, filaments glabrous. Ovary stalked, ca. 0.1–0.3 × 0.1–0.2 mm long, swollen; style ca. 0.3–0.6 × 0.03–0.04 mm long, sub-geniculate, covered with trichomes in two lines; stigma brush type and contracted, ca. 0.27 mm long, covered with trichomes; ovules up to 3. Pods light brown, obovate-oblong, ca. 0.5–0.9 × 0.2–0.4 cm, glabrous, beak absent, style not persistent. Seeds brownish, matt, reniform, glabrous, ca. 0.1–0.2 × 0.05–0.06 mm.

#### Phenology

Flowering December to–February, fruiting January to March

#### Distribution, habitat and ecology

*Crotalaria multibracteata* -grows in dry and rocky substratum of the Panhala region (Fig 5) situated on the Panhala-Alta range of Kolhapur district. It occurs in the ranges (Panhala-Alta range) of northernmost Kolhapur (Kolhapur District Gazetteer 2006).

The region is a stretch of typical grassland with rocky mountain slopes and forest edges. The temperature has a relatively narrow range between 10°C to 35°C. Mean relative humidity in summer (March-May) is up to 65%, it is 87% during wet weather (June–October) and 63% in winter (November–February) [46].

*Crotalaria multibracteata* has a sympatric distribution with *C*. *vestita*. Other plants associated with *C*. *multibracteata* include *Terminalia chebula* Retz., *Indigofera dalazelli* Cooke, *Erythyina stricta* Roxb., *Dalbergia latifolia* Roxb.

#### Etymology

The species is named for the multiple bracts (more than 4) present on the peduncle.

#### IUCN conservation status

Endangered (EN). The species is known only from the type locality. In accordance to the IUCN two [47–48] criteria, it should be best considered endangered according to the preliminary investigations made because it meets criterion of points A-E of section V of IUCN: (a) A suspected population size reduction of ≥50% over the last ten years, based on potential levels of exploitation of limestone and coal mining and jhum cultivation, and (b) Extent of occurrence suspected to be less than 5,000 km^2^ and known to exist in no more than five locations.

#### Species recognition

*Crotalaria multibracteata* is a procumbent slender herb with branched and terete stems. The species resembles *C*. *vestita* in having bi-lipped corolla, lanceolate and non-sticky calyx with acute apex, emarginate standard apex, glabrous keel surface, alae absent, beak twisted up to 90°, anther filament glabrous, gynoecium surface glabrous, style with two lined hairs, subgeniculate style curvature, pod surface glabrous, seed reniform and brown with glabrous surface (see Table 4). It differs from *C*. *vestita* in stem surface, leaf margin, petiole surface, calyx surface, extra bracts (more than 4) on peduncle, standard adaxial surface, keel shape, keel vestiture, number of seeds per pod and pod beak details of which are summarized below. *C*. *vestita* has densely cloth with yellow brown silky hairs (long and often bulbous based), stem surface whereas the newly discovered plant has velutinous stem with white hairs. Leaf margin is involute in case of *C*. *vestita* and simple in *C*. *multibracteata*. Petiole is less than 1 mm, densely hairy in *C*. *vestita* and sessile in *C*. *multibracteata*. Bract margin, bract shape and bracts on peduncle in *C*. *vestita* are non-ciliate, lanceolate or ovate-lanceolate, bracts equal to flower length compared to ciliate margins, linear and multiple bracts (more than 4) on the peduncle. Standard adaxial surface is glabrous in *C*. *vestita* and apex-pubescent in *C*. *multibracteata*. Keel shape and keel vestiture is angled and lanate vestiture (ciliate glabrous in *C*. *vestita*). Number of seeds per pod is 1–4 (vs.15-33 in *C*. *vestita*). Pod beak is absent in *C*. *multibracteata* (vs. present in *C*. *vestita*). A comparative morphology of all the characters between *Crotalaria multibracteata* and its close allies has been provided in Table 4. Based on the morphological and molecular evidences, the plant collected from Panhala, Kolhapur, is best placed in *Crotalaria* sect. *Calycinae* based on keel curvature, twisted beak, calyx more than half as long as the keel to longer than the keel, often bilipped and leaves usually simple.

**Table 4:** Diagnostic features and comparative morphology of *Crotalaria multibracteata* with its morphologically most similar species (based on Sanjappa 1991 [49], Ansari 2008 [10], Le Roux et al. 2013 [6], Subramaniam et al. 2013, 2015 [8, 15]; present study). Bold font represents the main distinguishing features of the new species with its close allies.

Characters	*Crotalaria multibracteata*	*Crotalaria vestita*
Habit	Procumbent slender herb	Erect or procumbent much branched herb
**Height**	**ca. 50 cm**	**ca. 40–90 cm**
Stem surface	Velutinous	Dense yellow brown silky hairs (long and often bulbous based)
**Leaf margin**	**Simple**	**Involute**
**Leaf size**	**1–1.5 x 0.4–0.7 cm**	**Up to 4 cm long**
**Petiole**	**Absent**	**Less than 0.1 cm**
Petiole surface	Not Applicable	Densely hairy
Leaf shape	Ovate-oblong	Oblong, oblong-lanceolate
Leaf apex	Abruptly acute	Subacute or obtuse at apex
**Leaf surface**	**Hirsute with ciliate margins**	**Hirsute with ciliate margins**
Inflorescence	Terminal or leaf opposed	Densely flowered, leaf opposed or terminal raceme
Inflorescence length	ca. 2–5 cm long	ca. 1.5–4.5 cm long
Number of flowers per inflorescence	2 to 4	4 to 5
Flower colour	Pale yellow	Greenish yellow
Flower size	0.6–0.75 x 0.35–0.45 cm	0.6–0.9 cm
**Calyx length**	**0.07–0.1 cm**	**0.65–0.9 x 0.1–0.2 cm**
Calyx surface	Pubescent paleaceous	Densely hairy
Calyx margin	Densely ciliate	Densely hairy
**Bract size**	**ca. 0.1cm**	**ca. 0.4–1.6 x 0.1–0.3 cm**
**Bracts on peduncle**	**Extra bracts (more than 4) on peduncle**	**Bracts equal to flower**
Bract shape	Linear	Lanceolate or ovate-lanceolate
Bract surface	Densely silky pubescent	Densely hairy, pilose, foliaceous
**Bract margin**	**Ciliate**	**Bract margin not ciliate**
Bracteole shape	Lanceolate	linear
Bracteole surface	Densely silky pubescent	Densely hairy-hirsute
Bracteole size	Up to 2 mm long	3–6 mm long
Standard shape	Obovate-orbicular	Sub-orbicular or orbicular-oblong
Standard dorsal surface	Apex-midvein pubescent	Glabrous
**Standard size**	**0.08–0.1 x 0.06–0.1 cm**	**0.65–0.9 x 0.3–0.6 cm**
Standard color	Light yellow	Greenish yellow
Wing size	0.03–0.1 x 0.01–0.04 cm	0.65–0.8 x 0.15–0.25 cm
Claw size	0.06–0.08 × 0.04–0.05	0.1–0.2 cm
Keel size	0.08–0.1 × 0.03–0.04 cm	0.6–0.8 x 0.6–0.7 cm
**Keel shape**	**Subangled**	**Angled**
**Keel curvature**	**Basal half**	**Lower third**
**Keel vestiture**	**Lanate vestiture**	**Ciliate glabrous**
Androecium size	Staminal sheath 0.02–0.05 cm long	Staminal sheath 0.01–0.05 cm long
	Filaments 0.13–0.17 cm long	Filaments 0.1–0.25 cm long
	Longer anther (oblong or linear) 0.01–0.09 cm long	Longer anther (oblong) *ca*. 0.1 cm long
	Shorter anther (ovoid) 0.02–0.04 cm long	Shorter anther (ovoid) *ca*. 0.02 cm long
Gynoecium size	00.1–0.03 x 0.01–0.02 cm	0.2–0.4 cm
Style length	0.03–0.06 x 0.003–0.004	0.3–0.5 cm
**Stigma surface**	**Brush type**	**Dilated**
Pod stalk	ca. 0.01–0.1 cm	0.01–0.09 cm
Pod shape	Obovate-oblong	Oblong
Pod size	0.5–0.9 x 0.2–0.4 cm	1.6 x 0.5–0.8 cm
**Number of seeds per pod**	**1 to 4**	**15 to 33**
Pod beak	Absent	Present
Seed size	.0.1–0.2 x 0.05–0.06 mm	ca. 0.15 cm

## Supporting information

S1 Appendix Plant accessions used for the molecular analyses of Indian *Crotalaria* along with their GenBank accession numbers. Voucher specimen numbers and locality details for collections are also provided along with the co-ordinates for their collection. All the specimens have been deposited in Delhi University Herbarium (DUH) and Munich Herbarium (M).
(DOC)

S2 Appendix Coordinates for plotting the distribution of *Crotalaria* species in Maharashtra state, India. (DOC)

S3 Appendix Cosine values for PCA. (XLSX)

## References

[1] Van Wyk BE. Tribe Crotalarieae In: Lewis G, Schrire B, Mackinder B, Lock M, editors. Legumes of the World. Royal Botanic Gardens Kew, Richmond, U.K.; 2005 pp. 21–28.

[2] Lewis G, Schrire B, Mackinder B, Lock M. Legumes of the World: Royal Botanical Gardens Kew, Richmond, U.K.; 2005.

[3] Boatwright JS, Le AT, Wink MM, Morozova T, van Wyk BE. Phylogenetic relationships of tribe Crotalarieae (Fabaceae) inferred from DNA sequences and morphology. Syst Bot. 2008; 33: 752–761. doi: 10.1600/036364408786500271

[4] Boatwright JS, Tilney PM, van Wyk BE. The generic concept of Lebeckia (Crotalarieae, Fabaceae): Reinstatement of the genus Calobota and the new genus Wiborgiella. S Afr J Bot. 2009; 75: 546–556. doi: 10.1016/j.sajb.2009.06.001

[5] Boatwright JS, Wink MM, Van Wyk BE. The generic concept of *Lotononis* (Crotalarieae, Fabaceae): Reinstatement of the genera *Euchlora*, *Leobordea* and *Listia* and the new genus *Ezoloba*. Taxon 2011; 60: 161–177.

[6] Le Roux MM, Boatwright JS, Van Wyk BE. A global infrageneric classification system for the genus *Crotalaria* (Leguminosae) based on molecular and morphological evidence. Taxon. 2013; 62: 957–971. doi: 10.12705/625.1

[7] Subramaniam S, Pandey AK. Taxonomy and phylogeny of the genus *Crotalaria* (Fabaceae), An overview. Acta Biolo Indica. 2014; 2(1): 253–264.

[8] Subramaniam S, Pandey AK, Geeta R, Mort ME. Molecular systematics of Indian *Crotalaria* (Fabaceae) based on analysis of nuclear ribosomal ITS DNA sequences. Plant Syst Evol. 2013; 299: 1089–1106. doi: 10.1007/s00606-013-0781-2

[9] Polhill RM. *Crotalaria* in Africa and Madagascar. Rotterdam: A.A. Balkema; 1982.

[10] Ansari AA. Crotalaria in India. Dehra Dun: Bishen Singh Mahendra Pal Singh; 2008.

[11] Flores AS, Correa AM, Forni Martins ER, Azevedo-Tozzi AMG. Chromosome numbers in Brazilian species of *Crotalaria* (Leguminosae, Papilionoideae) and their taxonomic significance. Bot J Linn Soc. 2006, 151: 271–277. doi: 10.1111/j.1095-8339.2006.00479.x

[12] Pandey A, Singh R, Sharma KS, Bhandari CD. Diversity assessment of useful *Crotalaria* species in India for plant genetic resources management. Genet Resour Crop Evol. 2010; 57: 461–470. doi: 10.1007/s10722-009-9517-0

[13] Eflora of Pakistan; 2017 [cited 2017 Oct 8]. Available from: http://www.efloras.org/florataxon.aspx?flora_id=5&taxon_id=10166

[14] Kumar S, Sane PV. Legumes of South Asia: A Checklist. Royal Botanic Gardens Kew 2003 pp.140–164.

[15] Subramaniam S, Pandey AK, Rather SA. A revised circumscription of the species in Bracteatae complex (section *Calycinae*) in the genus *Crotalaria*, Evidence from nuclear and chloroplast markers. Plant Syst Evol. 2015; 301: 2261–2290. doi: 10.1007/s00606-015-1228-8

[16] Danda S, Subramaniam S, Rather SA, Pandey AK. A New Species of *Crotalaria* (Fabaceae, Crotalarieae) from Meghalaya, India. Syst Bot. 2016; 41(2): 307–315. doi: 10.1600/036364416x691894

[17] Harris JG, Woolf Harris M. Plant Identification Terminology: an Illustrated Glossary. 2nd ed. Spring Lake Utah: Spring Lake Publishing; 2001.

[18] Hickey M, King C. The Cambridge illustrated glossary of Botanical terms: Cambridge University Press; 2007 doi: 10.1006/anbo.2001.1472

[19] Hewson H. Plant Indumentum: A handbook of terminology. Series no. 9. Canberra: Australian Government Publishing Service; 1990.

[20] Endress PK. Disentangling confusions in inflorescence morphology, patterns and diversity of reproductive shoot ramification in angiosperms. J Syst Evol. 2010; 48(4): 225–239. doi: 10.1111/j.1759-6831.2010.00087.x

[21] Thiers B. Index Herbariorum: A global directory of public herbaria and associated staff: New York Botanical Garden's Virtual Herbarium; 2016 http://sweetgum.nybg.org/ih/

[22] JSTOR Global Plants database; 2017 [cited 2017 Oct 20]. Available from: http://plants.jstor.org

[23] C Chinese Virtual Herbarium; 2017 [cited 2017 Nov 5]. Available from: https://primulaworld.blogspot.in/2015/12/the-chinese-virtual-herbarium-cvh.html

[24] Eflora of Pakistan; 2017 [cited 2017 Oct 8]. Available from: http://www.efloras.org/florataxon.aspx?flora_id=5&taxon_id=10166

[25] Hijmans RJ, Cameron SE, Parra JL, Jones PG, Jarvis A. Very high resolution interpolated climate surfaces for global land areas. Int J Climatol. 2005; 25: 1965–1978. doi: 10.1002/joc.1276

[26] ESRI Data and Maps for ArcGIS. 2002. Available at: https://www.lib.ncsu.edu/gis/esri2006.html

[27] White TJ, Bruns T, Lee S, Taylor J. Amplification and direct sequencing of fungal ribosomal RNA genes for phylogenetics In: Innas MA, Gelfand MA, Sninsky JJ, White TJ, editors. PCR Protocols. New York Academic Press; 1990 pp. 315–322. doi: 10.1016/b978-0-12-372180-8.50042–1

[28] Jing YU, Jian-Hua XUE, Shi-Liang ZHOU. New universal matK primers for DNA barcoding angiosperms. J Syst Evol. 2011; 49 (3): 176–181. doi: 10.1111/j.1759-6831.2011.00134.x

[29] Heracle BioSoft. DNA Sequence Assembler 4.36[Internet]. 2016. Available from: http://www.DnaBaser.com

[30] Thompson JD, Gibson TJ, Plewniak F, Jeanmougin F, Higgins DG. The ClustalX windows interface: flexible strategies for multiple sequence alignment aided by quality analyses tools. Nucleic Acids Res. 1997; 25: 4876–4882. doi: 10.1093/nar/25.24.4876 9396791 10.1093/nar/25.24.4876 PMC147148

[31] Katoh K, Standley DM. MAFFT multiple sequence alignment software 7: improvements in performance and usability. Mol Biol Evol. 2013; 30: 772–780. doi: 10.1093/molbev/mst010 23329690 10.1093/molbev/mst010 PMC3603318

[32] Katoh K, Standley DM. A simple method to control over-alignment in the MAFFT multiple sequence alignment program. Bioinformatics 2016; 32: 1933–1942. doi: 10.1093/bioinformatics/btw108 27153688 10.1093/bioinformatics/btw108 PMC4920119

[33] Maddison WP, Maddison DR. Mesquite 3.2. A modular system for evolutionary analysis. 2009 Available from: http://mesquiteproject.org

[34] Gene Codes Corporation. Sequencher 5.1 [Internet]. Available from: http://www.genecodes.com/

[35] Hopper SD, Fay MF, Rossetto M, Chase MW. A molecular phylogenetic analysis of the bloodroot and kangaroo paw family, Haemodoraceae; Taxonomic, biogeographic and conservation implications. Bot J Linn Soc. 1999; 131: 285–299. doi: 10.1111/j.1095-8339.1999.tb00770.x

[36] Chase MW, de Bruijn AY, Reeves G, Cox AV, Rudall PJ, Johnson MAT, Eguiarte LE. Phylogenetics of Asphodelaceae (Asparagales): an analysis of plastid *rbcL* and *trnL-F* DNA sequences. Ann Bot. 2000; 86: 935–956. doi: 10.1006/anbo.2000.1262

[37] Hodkinson TR, Chase MW, Takahashi C, Leitch IJ, Bennett MD, Renvoize SA. The use of DNA sequencing (ITS and *trnl-F*) AFLP and fluorescent in situ hybridisation to study allopolyploid *Miscanthus* (Poaceae). Am J Bot. 2002; 89: 279–286. doi: 10.3732/ajb.89.2.279 21669737 10.3732/ajb.89.2.279

[38] Akaike H. A new look at the statistical model identification. IEEE Trans Autom Contr. 1974; 19: 716–723.

[39] Guindon S, Gascuel O. A simple, fast and accurate algorithm to estimate large phylogenies by maximum Likelihood. Syst Biol. 2003; 52: 696–704. doi: 10.1093/sysbio/syq010 14530136 10.1080/10635150390235520

[40] Posada D. jModel test. Phylogenetic model averaging. Mol Bio Evol. 2008; 25: 1253–1256. doi: 10.1093/molbev/msn083 18397919 10.1093/molbev/msn083

[41] Ronquist F, Huelsenbeck JP. Mr BAYES 3: Bayesian phylogenetic inference under mixed models. Bioinformatics. 2003; 19: 1572–1574. doi: 10.1093/bioinformatics/btg180 12912839 10.1093/bioinformatics/btg180

[42] Stamatakis A. RAxML-VI-HPC: Maximum likelihood-based phylogenetic analyses with thousands of taxa and mixed models. Bioinformatics. 2006; 22: 2688–2690. doi: 10.1093/bioinformatics/btl446 16928733 10.1093/bioinformatics/btl446

[43] Scuri S, Torrisi M, Cocchioni M, Dell Uomo A. The European water framework directive 2000/60/EC in the evaluation of the ecological status of watercourses. Case study: the river Chienti (central Apennines, Italy). Acta Hydrochim Hydrobiol. 2006; 34: 498–505. doi: 10.1002/aheh.200600646

[44] McAleece N, Gage JDG, Lambshead PJD, Paterson GLJ. Bio Diversity professional statistics analysis software. Jointly developed by the Scottish Association for Marine Science and the Natural History Museum London; 1997.

[45] Kucharczyk H, Kucharczyk M. Application of PCA in taxonomy Research Thrips (Insecta, Thysanoptera) as a Model Group: Principal Component Analysis. Multidisciplinary Applications, Dr. Parinya Sanguansat (Ed.), In Tech; 2012. 10.5772/37602

[46] Government of Maharashtra. Gazetteer of Maharashtra State Kolhapur District: Gazetteer Department. Government of Maharashtra, Mumbai. 1989; p.107. Available from: http://kolhapur.nic.in/new/DistrictGazetteer/Home.html

[47] IUCN. Guidelines for Application of IUCN Red List Criteria at Regional and National Levels, version 4.0. Gland: Switzerland and Cambridge, UK: IUCN; 2012.

[48] IUCN. IUCN Red List of Threatened Species, version 2014.3. Gland: Switzerland and Cambridge, UK: IUCN; 2014.

[49] Sanjappa M. Legumes of India. Dehra Dun: Bishen Singh Mahendra Pal Singh; 1992.

