# Supplementary material for: Discovery of two new species of Crotalaria (Leguminosae, Crotalarieae) from Western Ghats, India
Source: PLoS One. 2018 Feb 15;13(2):e0192226. doi: 10.1371/journal.pone.0192226 (PMC5813922; doi:10.1371/journal.pone.0192226)
Supplement: S1 Appendix — Voucher specimen numbers and locality details for collections are also provided along with the co-ordinates for their collection. All the specimens have been deposited in Delhi University Herbarium (DUH) and Munich Herbarium (M). (DOC) [file pone.0192226.s001.doc]

**S1 Appendix. Plant accessions used for the molecular anlayses of Indian *Crotalaria* along with their GenBank accession numbers. Voucher specimen numbers and locality details for collections are also provided alongwith the co-ordinates for their collection. All the specimens have been deposited in Delhi University Herbarium (DUH) and Munich Herbarium (M).**

| **Taxon** | **DNA no.** | **Voucher (Herbarium)** | **Locality** | **Coordinate** | **ITS** | **matK** |
| --- | --- | --- | --- | --- | --- | --- |
| *Crotalaria agatiflora* Schweinf. | AKP 242 | Subramaniam 1020a | India, Tamil Nadu, Kodaikanal | 10.25272222,  77.50055556 | JN99021 | Submitted |
| *C. albida* Heyne ex Roth | AKP 283 | Subramaniam 903 | India, Uttarakhand, Dehradun | 30.308442, 78.074022 | JN990126 | Submitted |
| *C. albida* Heyne ex Roth | AKP 284 | Subramaniam 1058 | India, Tamil Nadu, Coonoor | 11.341775, 76.80505 | JN990120 | Submitted |
| *C. albida* Heyne ex Roth | AKP 285 | Subramaniam 1058a | India, Tamil Nadu, Coonoor | 11.359160,  76.799914 | JN990120 | Submitted |
| *C. angulata* Mill. | AKP 243 | Subramaniam 1086 | India, Tamil Nadu, Coimbatore | 11.000994, 76.939336 | JQ945951 | Submitted |
| *C. angulata* Mill. | AKP 244 | Subramaniam 1086a | India, Tamil Nadu, Coimbatore | 11.000994, 76.939336 | KP698615 | Submitted |
| *C. beddomeana* DC. | AKP 286 | Subramaniam 1083 | India, Kerala, Munnar | 10.03725000,  77.11861111 | JQ945935 | Submitted |
| *C. bifaria* Benth. | AKP 287 | Sharad Kamble SSK-1 | India, Karnataka, Badami | 15.917300,  75.673566 | JQ945936 | Submitted |
| *C. burhia* Benth. | AKP 245 | UdayBurman 22 | India, Rajasthan, Jodhpur | 26.255452,  72.992992 | JN990119 | Submitted |
| *C. berteroana* DC. | AKP 246 | Subramaniam 1073 | India, Kerala, Muthanga | 11.666576,  76.372579 | KR673341 | Submitted |
| *C. berteroana* DC. | AKP 247 | Subramaniam 1068 | India, Kerala, Muthanga |  | Submitted | Submitted |
| *C. calycina* Schrank | AKP 281 | Subramaniam 1006 | India, Tamil Nadu, Palani Hills | 10.360553, 77.406536 | KP698617 | Submitted |
| *C. calycina* Schrank | AKP 282 | Subramaniam 1011 | India, Tamil Nadu, Palani Hills | 10.357253, 77.412067 | JN990122 | Submitted |
| *C. clarkei* Gamble | AKP 288 | Subramaniam 1088 | India, Kerala, Wagamon | 9.74283333, 76.84777778 | JQ945932 | Submitted |
| *C. cytisoides* Roxb. ex DC. | AKP 248 | Rather Pandey 2706 | India, Arunachal Pradesh, BSI Campus | 27.819963  94.564023 | Submitted | Submitted |
| *C. cytisoides* Roxb. ex DC. | AKP 248 | Rather Pandey 2704 | India, Arunachal Pradesh, Doimukh | 27.14838889, 93.75055556 | Submitted | Submitted |
| *C. evolvuloides* Benth. | AKP 289 | Subramaniam 1049 | India, Tamil Nadu, Palani Hills | 10.39527778, 77.59222222 | KP698639 | Submitted |
| *C. epunctata* Dalz. | AKP 249 | Manudev 5202 | India, Odisha, Khandamal district | 20.176269, 84.086786 | JQ945952 | Submitted |
| *C. epunctata* Dalz. | AKP 250 | Manudev KM 5202a | India, Odisha, Khandamal district | 20.051856  83.910241 | JQ945952 | Submitted |
| *C. filipes* Benth. var. *trichophora* (Benth. ex Baker) Cook. | AKP 290 | Subramaniam and Pandey 3413 | India, Maharashtra, Kolhapur | 19.170531, 73.774261 | JQ945925 | Submitted |
| *C. filipes* var. *filipes* Benth. | AKP 291 | Subramaniam and Pandey 3416 | India, Maharashtra, Kolhapur | 18.090289, 74.263328 | JQ945926 | Submitted |
| *C. goreensis* Guill. & Perr. | AKP 292 | Subramaniam 1046 | India, Karnataka, Bengaluru, Yelahanka | 13.090558, 77.595594 | JN990128 | Submitted |
| *C. grahamiana* Wight & Arn. | AKP 293 | Subramaniam 1024 | India, Tamil Nadu, Pazhani hills | 10.445713,  77.508366 | JN990129 | Submitted |
| *C. hirta* Willd. | AKP 251 | Subramaniam 1092 | India, Tamil Nadu, Coimbatore | 11.04041667, 76.88083333 | JQ945928 | Submitted |
| *C. hirta* Rottl. ex Willd. | AKP 252 | Subramaniam Pandey 3423 | India, Maharashtra, Kohlapur | 16.699389,  74.83966 | JQ945929 | Submitted |
| *C. hirsuta* Willd. | AKP 296 | Subramaniam and Pandey 5026 | India, Himachal Pradesh | 42.61250000, 76.78777778 | KP698652 | Submitted |
| *C. hebecarpa* (DC.) Rudd | AKP 294 | Subramaniam 1031a | India, Tamil Nadu, Theni | 17.588358, 74.503647 | JN990130 | Submitted |
| *C. hebecarpa* (DC.) Rudd | AKP 295 | Subramaniam 3404 | India, Maharashtra, Kolhapur | 16.708309,  74.202861 | KP698610 | Submitted |
| *C. heyneana* Wight & Arn. | AKP 297 | Subramaniam 1061 | India, Kerala, Thamarassery pass | 11.49986111, 76.02194444 | JQ955942 | Submitted |
| *C. incana* L. | AKP 298 | Subramaniam 1008 | India, Karnataka, Bengaluru | 12.927122, 77.651419 | JN990131 | Submitted |
| *C. juncea* L. | AKP 253 | Subramaniam 1026 | India, Tamil Nadu, Kodaikanal | 10.224817, 77.483081 | JN990138 | Submitted |
| *C. juncea* L. | AKP 254 | Rather Pandey 2705 | India, Meghalaya, Nehu Campus | 25.60953056,  91.89944444 | Submitted | Submitted |
| *C. kurisumalayana* Sibichen & Nampy | AKP 299 | Subramaniam 1076 | India, Kerala, Kurisumalay | 9.681696,  76.887804 | KP698650 | Submitted |
| *C. Leschenaulti* DC. | AKP 300 | Subramaniam 1018 | India, Tamil Nadu, Palani Hills | 10.31022222, 77.56583333 | JN990118 | Submitted |
| *C. lutescens* Dalz. | AKP 255 | Subramaniam and Pandey 3434 | India, Maharashtra, Kolhapur | 16.813583, 74.119089 | JQ945934 | Submitted |
| *C. lutescens* Dalz. | AKP 256 | Subramaniam and Pandey 3417 | India, Maharashtra, Kohlapur | 16.695330,  74.175467 | JQ945933 | Submitted |
| *C. longipes* Wight & Arn. | AKP 257 | Subramaniam 1015 | India, Tamil Nadu, Palani Hills, Perumaalmalai | 10.3425, 77.576667 | KP698626 | Submitted |
| *Crotalaria longipes* Wight & Arn. | AKP 258 | Subramaniam and Pandey 1045 | India, Tamil Nadu, Shevaroy Hills | 11.855920,  78.217172 | JN990113 | Submitted |
| *C. meghalayensis* Danda and Pandey | AKP 301 | Danda and Pandey 1318 | India, Meghalaya, Jowai | 25.491944,  92.06138 | KR059588 | Submitted |
| *C. medicaginea* var. *luxurians* (Benth.) Baker | AKP 302 | Subramaniam 26 | India, Uttar Pradesh, Ghaziabad | 28.667917, 77.404961 | JN990115 | Submitted |
| *C. medicaginea* var. *luxurians* (Benth.) Baker | AKP 303 | Subramaniam 26a | India, Uttar Pradesh, Ghaziabad | 28.667917, 77.404961 | KP698668 | Submitted |
| *C. micans* Link | AKP 259 | Subramaniam 1051 | India, Tamil Nadu, Shevaroy Hills | 11.360944, 76.91925 | KP698660 | Submitted |
| *C. micans* Link | AKP 260 | Subramaniam 1057 | India, Tamil Nadu, Shevaroy Hills | 11.360944, 76.91925 | KP698659 | Submitted |
| *C. micans* Link | AKP 261 | Rather and Pandey 2719 | India, Arunachal Pradesh, Doimukh | 27.14847222,  93.75277778 | Submitted | Submitted |
| *C. mysorensis* Roth | AKP 304 | Subramaniam 1025 | India, Tamil Nadu, Kodaikanal | 10.268139, 77.588167 | JN990114 | Submitted |
| *C. obtecta* Wight & Arn. var. *obtecta* | AKP 305 | Subramaniam 1054 | India, Tamil Nadu, Coonoor | 11.357389, 76.796172 | KP698612 | Submitted |
| *C. obtecta var. glabrescens* (Benth.) Baker | AKP 306 | Subramaniam 1094 | India, Kerala, Munnar | 10.03725, 77.118639 | JQ945950 | Submitted |
| *C. orixensis* Willd. | AKP 307 | Subramaniam and Pandey 3429 | India, Maharashtra, Kolhapur | 16.677012,  74.324892 | JQ945923 | Submitted |
| *C. occulta* Grah. ex Benth. | AKP 308 | Shagun and Pandey 1319 | India, Meghalaya, Nehu Campus | 25.6106,  91.9014 | KR095590 | Submitted |
| *C. pallida* Ait. var. *pallida* (G. Don) Polhill | AKP 262 | Subramaniam 1066 | India, Karnataka, Bengaluru | 11.573167, 76.052944 | JQ945939 | Submitted |
| *C. pallida var obovata* (G. Don) Polhill | AKP 263 | Rather and Pandey 2709 | India, Arunachal Pradesh, Doimukh | 27.14847222,  93.75277778 | Submitted | Submitted |
| *C. pellita* DC. | AKP 309 | Subramaniam and Pandey 5072 | India, Andhra Pradesh, SK University campus | 14.618380,  77.655494 | KP698632 | Submitted |
| *C. pellita* DC. | AKP 310 | Subramaniam and Pandey 5081 | India, Tamil Nadu, Shevaroy Hills | 11.898876,  78.522760 | KP698633 | Submitted |
| *C. prostrata* Roxb. | AKP 313 | Subramaniam and Pandey 3428 | India, Karnataka, Belgaum | 15.808, 74.433736 | KP698616 | Submitted |
| *C. pulchra* Andr. | AKP 311 | Subramaniam and Pandey 5070 | India, Karnataka, Siddara Betta | 13.564890,  77.142111 | KX371741 | Submitted |
| *C. quinquefolia* L. | AKP 312 | Subramaniam 1060 | India, Kerala, Iringadampalli | 11.279972, 75.826111 | JQ945943 | Submitted |
| *C. retusa* L. | AKP 314 | Subramaniam 1005 | India, Delhi, Delhi University Botanical Garden | 28.688056, 77.211389 | KP698625 | Submitted |
| *C. semperflorens* Vent. | AKP 264 | Subramaniam 1055 | India, Tamil Nadu, Theni | 11.419611, 76.876889 | KP698674 | Submitted |
| *C. semperflorens* Vent. | AKP 265 | Subramaniam 1064a | India, Tamil Nadu, Theni | 11.419611, 76.876889 | JQ945940 | Submitted |
| *C. multibracteata* Rather & Pandey | AKP 274 | Rather and Pandey 2770 | India, Maharashtra, Kohlapur, Panhala | 16.81405000,  74.11888889 | **KY321450** | Submitted |
| *C. multibracteata* Rather & Pandey | AKP 275 | Rather and Pandey 2770a | India, Maharashtra, Kohlapur, Panhala | 16.81405000,  74.11888889 | **KY321451** | Submitted |
| *C.sessiliflora var sessiliflora* f. garhwalensis L. | AKP 280 | Subramaniam 5005 | India, Himachal Pradesh, Naahan | 30.72000000  77.42711111 | KP698619 | Submitted |
| *C. spectablis* Roth | AKP 315 | Subramaniam and Pandey 1099 | India, Delhi, Delhi University Botanical Garden | 28.688056, 77.211389 | JN990112 | Submitted |
| *C. stipitata* Wight & Arn. | AKP 272 | Subramaniam 1050 | India, Tamil Nadu, Ootacamund | 11.360194, 76.924583 | JN990116 | Submitted |
| *C. stipitata* Wight & Arn. | AKP 273 | Subramaniam 1071 | India, Tamil Nadu, Ootacamund | 11.360194, 76.924583 | KP698669 | Submitted |
| *C. suffruticosa* Subramaniam & Pandey | AKP 266 | Subramaniam and Pandey 3411 | India, Maharashtra, Kolhapur, | 16.5327777, 73.901388 | **KY321453** | Submitted |
| *C. suffruticosa* Subramaniam & Pandey | AKP 267 | Subramaniam and Pandey 3411a | India, Maharashtra, Kolhapur, | 16.5116666, 73.826111 | **KY321454** | Submitted |
| *C. suffruticosa* Subramaniam & Pandey | AKP 268 | Subramaniam and Pandey 3411b | India, Maharashtra, Kolhapur, | 16.5116666, 73.826111 | **KY321455** | Submitted |
| *C. suffruticosa* Subramaniam & Pandey | AKP 269 | Subramaniam and Pandey 3450 | India, Maharashtra, Kolhapur, | 16.5116666, 73.826111 | **KY321456** | Submitted |
| *C. salicifolia* Wight & Arn. | AKP 316 | Subramaniam 1079 | India, Kerala, Kurisumalay | 9.675372,  76.886883 | JQ945948 | Submitted |
| *C. tetragona* Andr. | AKP 317 | Pandey 10017 | India, Jammu and Kashmir, Jammu University Campus | 32.719334,  78.870070 | JN990110 | Submitted |
| *C. triquetra* Dalz. | AKP 278 | Subramaniam Pandey 3419 | India, Maharashtra, Kohlapur | 16.731466,  74.265087 | JQ945930 | Submitted |
| *C. verrucosa* L. | AKP 279 | Subramaniam 1014 | India, Tamil Nadu, Palani Hills | 28.620361, 77.177861 | KP698645 | Submitted |
| *C.vestita* Benth. | AKP 276 | Rather and Pandey 2780 | India, Maharashtra, Kohlapur, Panhala | 20.788343, 79.686038 | KY321452 | Submitted |
| *C. walkeri* Arn. | AKP 277 | Subramaniam 1037 | India, Tamil Nadu | 9.705917, 77.405111 | JN990111 | Submitted |
| *C. wightiana* Wight & Arn. | AKP 270 | Subramanim 1013 | India, Tamil Nadu, Pazhani hills | 10.34258333  77.57661111 | JQ945947 | Submitted |
| *C. wightiana* Wight & Arn. | AKP 271 | Subramaniam 1091 | India, Kerala, Munnar | 10.03725000  77.11863889 | JQ945954 | Submitted |
| *C. arenaria* [Benth***.***](http://www.theplantlist.org/tpl1.1/record/ild-5704) | - | Eden Foundation 65 | Niger, Zinder | - | AJ313485 | JQ067579 |
| *C. microcarpa* Benth. | - | Bidgood et al 3341 | Tanzania, Iringa | - | FJ839491 | JQ041079 |
| *C. virgultalis* DC. | - | Van Wyk MIR023 | South Africa, Northern Cape | - | JQ067143 | JQ041112 |
| *C. sphaerocarpa* DC. | - | Schutte MIR018 | South Africa, Limpopo | - | JQ067139 | JQ041106 |
| *C. multiflora* Arn. | - | Hepper 4590 | Sri Lanka, Uva | - | JQ067336 | - |
| *C. chinensis* L. | - | Sorensen et al 2254 | Thailand, Loei | - | JQ067335 | - |
| *C. incana* L. | - | Thulin et al 9114 | Somalia, Maakhir | - | JQ067262 | JQ041051 |
| *C. laburnifolia subsp petiolaris* (Franch.) Polhill | - | Thulin 10811 | Somalia, Sanaag | - | JX120578 | JX120589 |
| *C. lebeckioides* Bond | - | Le Roux et al 104 | South Africa, Western Cape | - | JQ067321 | JQ067568 |
| *C. sphaerocarpa* DC. | - | Le Roux et al 74 | Namibia, Otjozondjupa | - | JQ067308 | JQ067558 |
| *C. cistoides* DC. | - | Bidgood et al 4731 | Thailand, Kanchanaburi | - | JQ067188 | JQ041009 |
| *C. meyerana* Steud. | - | Williamson MlR015 | Namibia, Karas | - | JQ067136 | JQ041078 |
| *C. spartioides* DC. | - | Lutombi and Strohbach DL149 | Namibia, Hardap | - | JQ067149 | JQ041105 |
| *C. amoena* Baker | - | Bidgood Leliyo Vollesen MlR057 | Tanzania, Rukwa | - | JQ067167 | JQ040990 |
| *C. laeta* Mart. ex Benth. | - | Coradiv L7718 | Brazil, Ceara | - | JQ067325 | JQ067571 |
| *C. holosericea* Nees & Mart. | - | Lewis et al 933 | Brazil, Bahia | - | JQ067343 | JQ067572 |
| *C. laevigata* Lam. | - | MlR177 | Madagascar, Amoron’i Mania | - | - | JQ041065 |
| *C. monteiroi* Baker f. | - | Schutte 83 | South Africa, KwaZulu Natal | - | JQ067129 | JQ041083 |
| *C. recta* Rich. | - | Van Wyk 1844 | South Africa, Mpumalanga | - | JQ067135 | JQ041103 |
| *Euchlora hirsuta* (Thunb.) Druce | - | Boatwright 223 | South Africa, Northern Cape | - | EU347879 | JQ041113 |
| *Bolusia amboensis* (Schinz) Harms | - | Boatwright Le Roux Van Wyk Mannheimer MIR112 | South Africa, Northern Cape | - | EU347881 | JQ040984 |
