# Supplementary material for: Discovery of two new species of Crotalaria (Leguminosae, Crotalarieae) from Western Ghats, India
Source: PLoS One. 2018 Feb 15;13(2):e0192226. doi: 10.1371/journal.pone.0192226 (PMC5813922; doi:10.1371/journal.pone.0192226)
Supplement: S2 Appendix — (DOC) [file pone.0192226.s002.doc]

**S2 Appendix. Coordinates for plotting the distribution of *Crotalaria* species in Maharashtra state, India**

| Taxon | Latitude | Longitude | Locality |
| --- | --- | --- | --- |
| *C. acicularis* Buch.-Ham. | 16.513638 | 73.825255 | Kolhapur |
| *C. acicularis* Buch.-Ham. | 17.726564 | 73.820181 | Kass Plateau |
| *C. albida* Heyne ex. Roth. var. *albida* | 19.047126 | 72.898463 | Bombay |
| *C. albida* Heyne ex. Roth. var. *kangrensis* | 19.04927 | 72.897969 | Bombay |
| *C. berteroana* DC. var. *berteroana* | 18.281697 | 73.973833 | Pune |
| *C. berteroana* DC. var. *yasminii* (Almeida & Almeida) Ansari | 18.280686 | 73.974106 | Pune |
| *C. bifaria* L. | 16.810078 | 74.109869 | Kohlapur |
| *C. calycina* Schrank | 18.954083 | 72.814969 | Bombay |
| *C. calycina* Schrank | 17.649697 | 74.045672 | satara district |
| *C. calycina* Schrank | 17.719725 | 73.823367 | Satara |
| *C. chinensis* L. | 16.807803 | 74.117947 | Bombay |
| *C. chinensis* L. | 18.573003 | 73.777439 | Bombay presidency |
| *C. clavata* Wight & Arn. | 17.720381 | 73.810503 | Kohlapur |
| *C. filipes* Benth. var. *filipes* | 16.809978 | 74.108306 | Kohlapur district |
| *C. filipes* Benth. var. *filipes* | 16.990294 | 73.690547 | Pune district |
| *C. filipes* Benth. var. *filipes* | 18.747728 | 73.403431 | Pune district |
| *C. filipes* Benth. var. *filipes* | 18.090289 | 74.263328 | Pune district |
| *C. filipes* Benth. var. *filipes* | 18.766442 | 73.430753 | Pune district |
| *C. filipes* Benth. var. *filipes* | 19.472728 | 72.976603 | Thana district |
| *C. filipes* Benth. var. *filipes* | 19.773592 | 77.134764 | Thana district |
| *C. filipes* Benth. var. *filipes* | 19.934253 | 73.339956 | Thana district |
| *C. filipes* Benth. var. *filipes* | 19.114408 | 73.733636 | Pune district |
| *C. filipes* Benth. var. *filipes* | 18.47545 | 73.854414 | Pune district |
| *C. filipes* Benth. var. *filipes* | 18.475942 | 73.852936 | Pune district |
| *C. filipes* Benth. var. *filipes* | 18.164231 | 74.093913 | Pune district |
| *C. filipes* Benth. var. *filipes* | 16.415233 | 73.998461 | Kohlapur district |
| *C. filipes* Benth. var. *filipes* | 19.119622 | 72.850686 | Solapur district |
| *C. filipes* Benth. var. *filipes* | 19.724308 | 75.134619 | Solapur district |
| *C. filipes* Benth. var. *filipes* | 70.680314 | 74.015792 | Satara district |
| *C. filipes* Benth. var. *filipes* | 15.937253 | 73.765042 | Ratnagiri district |
| *C. filipes* Benth. var. *filipes* | 21.402247 | 77.313214 | India, Maharshtra, Haripura district |
| *C. filipes* Benth. var. *filipes* | 21.260544 | 75.695811 | Jalgaon district |
| *C. filipes* Benth. var. *filipes* | 20.182856 | 73.328553 | Nasik district |
| *C. filipes* Benth. var. *trichophora* Benth. ex Baker | 19.170531 | 73.774261 | Pune district |
| *C. filipes* Benth. var. *panthakii*  Almeida & Almeida | 18.515281 | 73.839225 | Pune district |
| *C. hirsuta* Willd. | 17.661997 | 74.363608 | Satara district |
| *C. hebecarpa* (DC.) Rudd. | 17.588358 | 74.503647 | Satara district |
| *C. hirta* Rottl. ex Willd. | 17.833436 | 73.330086 | Palghar district |
| *C. hirta* Rottl. ex Willd. | 16.589483 | 74.312617 | Kohlapur district |
| *C. hirta* Rottl. ex Willd. | 16.679972 | 74.254439 | Kohlapur district |
| *C. juncea* L. | 17.720683 | 73.819519 | Satara district |
| *C. leptostachya* Benth. | 17.622192 | 73.861397 | Kohlapur district |
| *C. leptostachya* Benth. | 16.126383 | 73.946181 | Kohlapur district |
| *C. leptostachya* Benth. | 18.606122 | 73.798758 | Pune district |
| *C. leptostachya* Benth. | 18.767811 | 73.374539 | Khandala district |
| *C. leptostachya* Benth. | 19.150758 | 72.881992 | Bombay |
| *C. leptostachya* Benth. | 19.475292 | 72.974842 | Thana district |
| *C. leschenaultii* DC. | 17.929192 | 73.645986 | Bombay |
| *C. leschenaultii* DC. | 19.154653 | 73.174243 | Bombay |
| *C. linifolia* L.f. | 17.710014 | 74.319261 | Kohlapur district |
| *C. linifolia* L.f. | 17.649758 | 74.045928 | Satara district |
| *C. lutescens* Dalz. | 16.813583 | 74.119089 | Kohlapur district |
| *C. lutescens* Dalz. | 15.79405 | 74.315472 | Kohlapur district |
| *C. lutescens* Dalz. | 19.624678 | 79.80645 | Karwar district |
| *C. medicaginea* Lam. var. *medicaginea* | 19.768917 | 74.612636 | Kohlapur district |
| *C. medicaginea* Lam. var. *luxurians* (Benth.) Baker | 16.625103 | 74.373889 | Kohlapur district |
| *C. medicaginea* Lam. var. *luxurians* (Benth.) Baker | 17.682692 | 73.950589 | Kohlapur district |
| *C. medicaginea* Lam. var. *luxurians* (Benth.) Baker | 16.682992 | 74.255119 | Kohlapur district |
| *C. montana* Heyne ex Roth. | 16.793356 | 74.421569 | Kohlapur distruct |
| *C. montana* Heyne ex Roth. | 21.155675 | 79.049842 | Nagpur district |
| *C. multibracteata* Rather & Pandey | 16.818738 | 74.107926 | Kolhapur district |
| *C. mysorensis* Roth. | 16.541536 | 73.830353 | Kohlapur distruct |
| *C. mysorensis* Roth. | 17.721853 | 73.823058 | Sillagad district |
| *C. mysorensis* Roth. | 19.227036 | 72.929697 | Thane district |
| *C. mysorensis* Roth. | 20.305594 | 75.646847 | Sillod district |
| *C. mysorensis* Roth. | 19.475219 | 72.979497 | Thane district |
| *C. mysorensis* Roth. | 19.493175 | 72.859753 | Thane district |
| *C. mysorensis* Roth. | 20.903156 | 74.768764 | West Khandesh |
| *C. mysorensis* Roth. | 16.679411 | 74.256631 | Kohlapur district |
| *C. nana* Burm. f. | 16.676158 | 74.256525 | Kohlapur district |
| *C. nana* Burm. f. | 17.597792 | 73.848494 | Satara district |
| *C. nana* Burm. f. | 19.776144 | 74.988178 | Kohlapur district |
| *C. notonii* Wight & Arn. | 16.680322 | 74.168777 | India, Maharshtra |
| *C. orixensis* Rottl. ex Willd. | 18.537822 | 73.224464 | Pune district |
| *C. orixensis* Rottl. ex Willd. | 20.186742 | 79.991258 | Yavatamal district |
| *C. orixensis* Rottl. ex Willd. | 20.478103 | 77.480181 | Nagpur district |
| *C. orixensis* Rottl. ex Willd. | 20.975225 | 80.164933 | Jalgoan district |
| *C. orixensis* Rottl. ex Willd. | 17.969969 | 73.863225 | Satara district |
| *C. orixensis* Rottl. ex Willd. | 18.608689 | 72.896703 | Satara district |
| *C. orixensis* Rottl. ex Willd. | 18.959614 | 72.836306 | Nasik district |
| *C. pallida* Ait. var. *pallida* | 19.517541 | 74.277201 | Kolhapur |
| *C. pallida* Ait. var. *pallida* | 17.652748 | 75.899711 | Nasik |
| *C. pallida* Ait. var. *pallida* | 19.989038 | 73.783889 | Nasik |
| *C. pallida* Ait. var. *pallida* | 19.997603 | 73.846135 | Solapur |
| *C. pallida* Ait. var. *obovata* (G. Don.) Polhill | 21.139652 | 79.084605 | Kolhapur |
| *C. pallida* Ait. var. *obovata* (G. Don.) Polhill | 21.138652 | 79.0819 | Kolhapur |
| *C. prisetleyoides* Benth. ex Baker | 17.238958 | 73.958506 | India, Maharshtra |
| C. prostrata var. *prostata* Rottl. ex Willd. | 16.802378 | 74.119072 | India, Maharshtra |
| *C. prostrata* var. *prostata* Rottll. ex Willd. | 16.9997 | 73.319117 | India, Maharshtra |
| *C. prostrata* var. *prostata* Rottll. ex Willd. | 19.477239 | 72.978536 | India, Maharshtra |
| *C. pulchra* Andr. | 18.520431 | 73.856744 | Pune district |
| *C. pusilla* Heyne ex Roth. | 19.761806 | 74.995617 | Kohlapur district |
| *C. pusilla* Heyne ex Roth. | 16.683322 | 74.255383 | Kohlapur district |
| *C. pusilla* Heyne ex Roth. | 17.721064 | 73.819022 | Satara distruct |
| *C. pellita* Bert. ex DC. | 16.66295 | 74.0827 | India, Maharshtra |
| *C. retusa* L. | 17.723261 | 73.817503 | Satara district |
| *C. spectabilis* Roth. | 17.719925 | 73.818628 | Satara district |
| *C. stocksii* Benth. ex Baker | 19.783347 | 79.153553 | Chanderpur district |
| *C. suffruticosa* Subramaniam & Pandey | 16.5327778 | 73.901388 | Kolhapur district |
| *C. suffruticosa* Subramaniam & Pandey | 16.5116667 | 73.826111 | Kolhapur district |
| *C. trifoliastrum* Willd. | 18.541203 | 73.856744 | Pune district |
| *C. triquetra* Dalz. | 17.164758 | 73.900708 | Satara district |
| *C. triquetra* Dalz. | 18.501153 | 73.513644 | Pune district |
| *C. triquetra* Dalz. | 18.255325 | 73.815958 | Pune district |
| *C. triquetra* Dalz. | 19.791864 | 72.983886 | Thana district |
| *C. triquetra* Dalz. | 18.619346 | 73.375998 | Pune district |
| *C. triquetra* Dalz. | 18.617383 | 73.375367 | Pune district |
| *C. verrucosa* L. | 17.584766 | 74.499616 | Satara district |
| *C. verrucosa* L. | 16.818541 | 74.096006 | Kolhapur district |
| *C. vestita* Baker | 20.788343 | 79.686038 | Pune district |
